# In vivo and in vitro antidiabetic effects of phlorizin and its green-synthesized phlorizin-selenium nanoparticles in male rats: mechanistic involvements and nanoparticles characterization

**DOI:** 10.1186/s40643-026-01034-3

**Published:** 2026-04-27

**Authors:** Elshahat A. Toson, Entsar A. Saad, Mohammad M. Mashaly, Hadeer A. Omar

**Affiliations:** Chemistry Department, Faculty of Science, Damietta University, Damietta, New-Damietta, Egypt

**Keywords:** Diabetes, Phlorizin, PHZ-SeNPs, Adiponectin, Leptin, HOMA-IR

## Abstract

Selenium nanoparticles (Se NPs) play a substantial role in human body. Therefore, they were newly and greenly synthesized by using phlorizin (PHZ). The antidiabetic effects of PHZ and its greenly synthesized PHZ-SeNPs were tested in STZ-induced diabetes. The PHZ-SeNPs were red in color, UV-visible spectrophotometry (λmax 322 nm). Infrared spectroscopy verifies the production of Se NPs' coated with PHZ. TEM image represents spherically shaped particles (smaller average size, 5-11 nm). Zeta potential was -28.77 ± 9.5 mV with size distribution of 395 nm for PHZ-SeNPs. The loading capacity (LC) and encapsulation efficiency (EE) were 42.8% and 38.96%, respectively. FBG, HbA1c, calculating HOMA-IR, oral glucose tolerance test as well as insulin, adiponectin (ADP) and leptin using anti-rat were done. In addition, Leptin/Adiponectin ratio (LAR) were calculated. The LD_50_ of PHZ-SeNPs was 173.68 mg/kg. FBG level of diabetic rats was 477.67 ± 30.4 mg/dL, that after PHZ-SeNPs treatment was 206.83 ± 34.69 mg/dL and that of insulin-treated rats (237.17 ± 54.04 mg/dL). HOMA-IR levels were positively and significantly correlated with FBG, insulin level, lipid profile; expect HDL and that of leptin levels but were negatively and significantly correlated with ADP levels, and LAR. In addition, diabetes reduces liver content of glycogen which was elevated after treatments. As an in vitro study, IC_50_ of free PHZ was found to be 4.87 mM which was reduced up to 3.28 mM if PHZ-SeNPs were used (DPPH assay). The treatments improve liver, kidney and pancreatic histopathological finding. In conclusion: PHZ-SeNPs can be usefully used to reduce blood sugar and prevent diabetic complications. The mechanisms may involve both the intrinsic radical scavenging activity of PHZ and PHZ-SeNPs (as evidenced by DPPH assay) and the enhancement of endogenous antioxidant defenses (increased reduced glutathione, superoxide dismutase, and catalase levels), in addition to improved insulin secretion, ADP stimulation, leptin inhibition and increment in erythrocytes-glucose uptake.

**Supplementary Information:**

The online version contains supplementary material available at 10.1186/s40643-026-01034-3.

## Introduction

Diabetes mellitus is a chronic condition characterized by elevation in serum glucose concentrations higher than the normal threshold. It is two forms; type 1 (5%) and type 2 (90% to 95%). Regretfully, there is a growing chance of kidney, eye, and blood vessel damage with time. These can indirectly impact the heart (Verma et al. 2025).

Type 1 diabetes is autoimmune disease in which the immune system targets the pancreatic cells with subsequent hypoinsulinemia, hyperglycemia, and an elevated risk of ketonemia following the injury and/or destruction of 80% of the pancreatic beta cells (Arivarasan et al. 2025). Conversely, insulin deficiency as well as its resistance causes type 2 diabetes (T2D). This kind is marked by the gradual onset of insulin resistance and malfunctioning of β-cells (Mateo-Gavira et al. 2021). Neuropathy and diabetic microvascular consequences (retinopathy, nephropathy) are qualitatively comparable in both forms of diabetes. Additionally, the risk of atherosclerotic macrovascular problems is increased by both forms of diabetes (Arivarasan et al. 2025). Streptozotocin (STZ), a diabetic inducer, was utilized in the experiments to cause diabetes through selective toxicity to pancreatic β-cells, leading to impaired insulin secretion and hyperglycemia (Koroglu et al. 2025).

Phlorizin (PHZ) is a glucoside derivative of phloretin, a phenolic phytoconstituent that is a member of the bicyclic flavonoids’ family. It was separated from the apple tree’s bark. Phloridzin, phloretin 20-O-glucoside, phlorhizin, and phlorizoside are some of its common names. Many pharmacological actions of these chemicals have been demonstrated; the most researched ones are its antidiabetic activities. It dissolves in hot water and ethanol. PHZ is the first substance identified as a sodium-glucose co transporter (SGLT) antagonist, also improves insulin sensitivity (Khanam et al. 2022).

Selenium (Se) is a trace mineral. It has fundamental importance to human health as it is essential element in our bodies. Numerous stable organic selenium compounds with anti-tumor, cytokine-inducing, enzyme-inhibiting, and immunomodulatory properties have been created (Ullah et al. 2021). Se NPs can be made using both oxidation and reduction methods. For these reasons, phlorizin as a single and straight forward photochemical was used to assess in the green synthesis of PHZ-SeNPs. Their potential antidiabetic application will be evaluated. Abdel Moneim et al. (2015) create environmentally friendly non-materials that have more benefits than chemically manufactured ones. Se NPs’ enormous surface–volume ratio, high surface energy, and spatial constraint provide them unique chemical and physical characteristics (Lin et al. 2021).

Adipose tissues release a cytokine called adiponectin (ADP). This cytokine correlate with inflammation, lipid accumulation, and insulin sensitization modification (Achari and Jain 2017). Furthermore, the hypothalamus contains the primary neuronal targets of leptin. Therefore, it appears contradictory that while leptin’s circulating levels falls during fasting, it stimulates lipolysis in adipose tissue (Caron et al. 2018).

For the previous reasons, this work combined both in vivo and in vitro approaches to evaluate the antidiabetic potential of PHZ and PHZ-SeNPs. The in vivo part assessed their therapeutic effects against STZ-induced diabetes in rats, whereas the in vitro experiments on RBCs from normal and diabetic rats aimed to investigate their direct impact on glucose uptake. Future studies in non-diabetic animals are recommended to clarify the independent effects of PHZ and PHZ-SeNPs, separate from those induced by STZ.

## Materials and methods

### Chemicals

Chemicals and reagents used in this study were obtained from Sigma-Aldrich (USA) and Merck-Sigma (Steinheim, Germany), including streptozotocin (STZ). Sodium selenite (Na_2_SeO_3_) was employed as a selenium precursor in the manufacture of Se NPs. Act rapid insulin (1 ml contain 100 IU human insulin) was obtained from Novo Nordisk, Egypt. We bought phlorizin dihydrate from Sigma-Aldrich Co. in St. Louis, Missouri, USA. Merck (Darmstadt, Germany) provided us with the 2, 2- diphenyl-1-picryl hydrazyl-hydrate free radicals (DPPH). All biochemical parameters were measured using commercial diagnostic kits supplied by Bio diagnostic Co. (Cairo, Egypt), following the manufacturer’s protocols. All absorbance readings were recorded using spectrophotometer (JASCO V-630, Japan). Serum levels of albumin (CAT. NO. AB1010) and total protein (CAT. NO. TP2020), as well as the activities of alanine transaminase (ALT) (CAT. NO. AL1031(45) and aspartate transaminase (AST) (CAT. NO. AS1061(45) were evaluated by colorimetric/endpoint method (Gornall et al. 1949; Reitman and Frankel 1957; Doumas et al. 1971). The level of serum creatinine (CAT. NO. CR1251) was measured by non-enzymatic, colorimetric/kinetic method but urea (CAT. NO. UR2110), and uric acid (CAT. NO. UA2120) were measured by enzymatic, colorimetric/endpoint method (Chaney and Marbach 1962; Bartels et al. 1972; Zhao et al. 2009). Lipid profile parameters were measured by colorimetric/endpoint method (total cholesterol (CAT. NO. CH1220), triglycerides (CAT. NO. TR2030), HDL-cholesterol (CAT. NO. CH1230), total lipid (CAT. NO. TL2010)). As directed by the manufacturer, serum levels of insulin (CAT. NO. ELK2370), ADP (CAT. NO. ELK2463), and leptin (CAT. NO. ELK1244) were measured using their anti-rats (ELISA kits from ELK Biotechnology CO. Ltd., China) by Awareness Stat Fax ChroMate 4300, Awareness Technology Inc., USA.

### Preparation of selenium nanoparticles

For the green synthesis of Se NPs in the current investigation, PHZ reduce sodium selenite, as described by Sentkowska and Pyrzyńska (2023). Briefly, 2.5 mL of selenium solution (0.1 mol L^− 1^, 0.0432 g) was placed on the stirrer, followed by 15 mL of deionized water. Then, 2.5 mL of PHZ (0.1 mol L^− 1^, 0.1181 g) was added dropwise under continuous stirring at 150 R.P.M. and 37 °C for 12 h. The obtained nanoparticles were so strongly stabilized making it impossible to isolate them from the post-reaction mixture using e.g., centrifugation.

### Characterization of Se NPs

The generated nanoparticles’ physical characteristics and chemical make-up, including their size, nature of the surface, structure and morphology information were determined using a transmission electron microscopy (TEM) (Electron Microscope Unit, Alex University, Egypt, JEOL TEM-2100, Tokyo, Japan). The functional groups of the synthesized NPs were characterized using a JASCO FT/IR-4100 spectrometer (Japan) at the Faculty of Science, Damietta University, Egypt, scanning within the range of 4000 to 400 cm^− 1^. UV–Vis spectrophotometer (JASCO V-630) using double beam spectrum was used to investigate the optical characteristics of Se NPs. The average hydrodynamic size and zeta potential and the poly dispersity index (PDI) were determined using Zetasizer Nano-ZS90 software (Version 2.3). The surface energy of the produced Se NPs in solution was measured using the zeta potential method (Kassel, Germany) in Damietta University, Egypt.

### Determination of loading capacity (LC) and encapsulation efficiency (EE)

Direct method was used to determine PHZ encapsulation in the nanoparticles (Garms et al. 2021). The PHZ concentration is calculated by the standard curve. The content of PHZ in the nanoparticles was determined by a UV spectrophotometer at λmax = 284 nm according to the following equations:

$${\mathrm{LC}}\% ={\text{ }}{{\mathrm{m}}_{{\text{PHZ }}({\mathrm{e}})}}{\mathrm{/}}{{\mathrm{m}}_{{\mathrm{NP}}}}*{\mathrm{100}}$$

$${\mathrm{EE}}\% ={\text{ }}{{\mathrm{m}}_{{\text{PHZ }}({\mathrm{e}})}}{\mathrm{/}}{{\mathrm{m}}_{{\mathrm{PHZ}}}}*{\mathrm{1}}00$$

Symbols used: m _PHZ_ = mass of PHZ initially used, m _PHZ(e)_ = mass of PHZ (encapsulated), m _NP_ = mass of dry NPs.

### Assessment of the antioxidants activity of PHZ and PHZ-SeNPs using DPPH assay

DPPH assay was used to assess the electron- or hydrogen-donating ability of PHZ and PHZ-SeNPs and their radical scavenging activity. Among the most useful property of such methods is the detection of antioxidant potential of the target (Huguet-Casquero et al. 2020).

### Estimation of erythrocytes-glucose uptake

Using the Jain technique, erythrocytes from diabetic rats and controls were three times washed with phosphate buffered saline (PBS). The washed cells were inoculated with 350 mg glucose/dL PBS for two hours at 37 °C (Jain 1989).The ability of the cleaned erythrocytes to absorb glucose from a PBS solution with a specific glucose concentration was assessed. By subtracting glucose’s optical density acquired after (final) incubation from that obtained before (initial) incubation, the amount of remaining (unconsumed) glucose can be calculated.

$$\% {\text{ of glucose uptake value}}={(Initial glucose - Final glucose) / Initial glucose {X 1}}00$$

### Animals

All procedures were conducted in accordance with the National Institutes of Health (NIH) guidelines. Male albino rats of the Wistar strain (8–10 weeks old, 150–180 g at the start of the study) were acquired from the animal station in Abo Rawash, Egypt, and allowed to acclimate for 14 days at a temperature of 25 ± 3.2 °C with 12/12-hour cycles of light and dark. Every rat was housed in a hygienic polypropylene cage and given unlimited access to food and drink. All animal experiments are carried out according to the Guide for the Care and Use of Laboratory Animals, according to the IACUC, approved by the Institutional Review Board of the Faculty of Medicine, Al-Azhar University (No. DFM-IRB 00012367-25-07-022, Issuing: 10 August 2025).

### Methodological design

The male rats received a single dose of STZ via intraperitoneal injection (i.p.) (Sigma, Chemical) following the 14-day acclimation period. STZ was freshly prepared immediately prior to administration, and the solutions were kept on ice and protected from light. STZ (50 mg/kg) was dissolved in sodium citrate buffer (0.1 mol/liter, pH 4.5). On the day of STZ administration, rats were provided with 5% glucose solution instead of regular drinking water to prevent severe hypoglycemia. Diabetes was induced in the rats by a single intraperitoneal injection of STZ, which causes progressive β-cell dysfunction and injury. Blood glucose levels were monitored daily using a blood glucose meter (Precichek AC-302, auto code series, Germany), with blood samples obtained from the tail vein. Rats exhibiting blood glucose levels above 300 mg/dL within three to five days after the injection were selected for the subsequent study and received daily treatments for 4 weeks. Five groups of six rats each were randomly selected from among the animals. G1: Healthy control group and was fed only regular food. G2: Diabetic control (50 mg/kg) i.p single injection with STZ, G3: Insulin-treated group (50 mg/kg) STZ, single i.p and subcutaneously treated with 4 IU/Kg insulin. G4: PHZ-treated group (50 mg/kg STZ, single i.p) followed by intragastric gavage treat with 100 mg/Kg of PHZ. G5: PHZ-SeNPs-treated group (50 mg/kg STZ, single i.p). followed by i.p. PHZ-SeNPs treatment (10 mg/kg) which equivalent 4.28 mg/kg PHZ.

### Determination of LD_50_ of PHZ-SeNPs

In order to determine the median lethal dose (LD_50_) PHZ-SeNPs, male rats were used according to the method as was previously described by (Akçay 2013).

### Samples

After fasting for 6–8 h, tail vein blood was used to estimate FBG. Further, after 1 and 2 h of meal intake, another blood samples obtained to estimate random and post prandial blood sugar, respectively. Ultimately, all animals were fasted for 12 h and then euthanized under deep isoflurane anesthesia upon completion of the 30-day experimental period. Clean, dry tubes containing or lacking EDTA were used to collect whole blood. Following a 15-minute centrifugation at 4000 r.p.m., the sample sera were separated and stored at -20 °C for the biochemical parameter tests later.

### Glucose tolerance test (GTT)

The glucose tolerance test (GTT) was performed during the final week of the treatment period, 24 h prior to euthanasia, according to the method previously described (Mei et al. 2016). Briefly, animals were fasting for 8 h, then received Glucose (2 g/kg) orally by gavage. Blood samples were collected via the tail vein at 0, 60, and 120 min for glucose measurement after glucose loading (Meighan et al. 2025). Glucose tolerance was assessed after administration of i.p. PHZ-SeNPs and Sc. for insulin to the diabetic rats. On the other hand, PHZ was orally administered. The routes of administration were selected based on the physicochemical properties and stability of each compound (Garms et al. 2021). 3 blood samples were taken to construct this glucose tolerance curve by the method of (Trinder 1969). GraphPad Prism 6.0 (La Jolla, CA, USA) was used to compute area under the curve (AUC) for this two-hour period.

### Determination of oxidative stress biomarkers in liver tissues

A known weight of rat’s liver tissues was washed 3 times with normal saline. Then, this tissue weights were mixed together in a 50 mM, pH 7.5 ice-cold phosphate buffer. In a cooling centrifuge, the resulting homogenate (10%, w/v) was centrifuged for 20 min. at 4 °C at 12,000 r.p.m. After that, upper layer was gathered and kept for use in ensuing biochemical and antioxidant tests at -20 °C. The enzymatic activities of superoxide dismutase (SOD, CAT. NO. SD2521) was measured by enzymatic, colorimetric/kinetic method while catalase (CAT, CAT. NO. CA2517) were determined by enzymatic, colorimetric/endpoint method (Sinha 1972; Paoletti et al. 1986). Also, reduced glutathione (GSH, CAT. NO. GR2511), malondialdehyde (MDA, CAT. NO. MAK568), nitric oxide (NO, CAT. NO. NO2533) and total antioxidant capacity (TAC, CAT. NO. TA2513) were evaluated by colorimetric/endpoint method (Uchiyama and Mihara 1978; Koracevic 2001; Rahman et al. 2006; Menaka et al. 2009).

### Biochemical assessment of liver glycogen

With minor adjustments, the Carroll technique was used to calculate the hepatic glycogen concentration (Carroll et al. 1956). Briefly, glycogen was extracted from liver tissue by boiling with 30% KOH, and determine after alcohol precipitation. The precipitate was treated with phenol and sulfuric acid for quantification instead of anthrone reagent. The calibration curve was constructed using glycogen (Sigma) as the standard. The amount of glycogen was measured in milligrams per gram of liver tissue.

### Histological studies

The pancreatic, kidney, and liver tissue samples were kept in 10% neutral formalin buffered. They gradually dried cleaned in ethanol (70%) and xylene. After that they infiltrated with paraffin wax. Hematoxylin & eosin dyes were used to stain 5 μm paraffin sections for microscopic inspection. Also, liver tissues were stained by periodic acid–Schiff (PAS) for glycogen contents detection in all animals’ groups using the method of Hui et al. (2017). A semi-quantitative assessment was conducted by counting the number of islets per 10 high-power fields (HPFs) and estimating the number of cells per islet under light microscopy.

### Statistical analyses

The mean ± S.D. was used to express the data. ANOVA followed by LSD post hoc test was used to compare differences between groups. *P* ≤ 0.05 was regarded as a statistically significant probability value. Version 20 of SPSS was used to analyze the data.

## Results

### Characterization of PHZ and PHZ-SeNPs

**Fig. 1:**
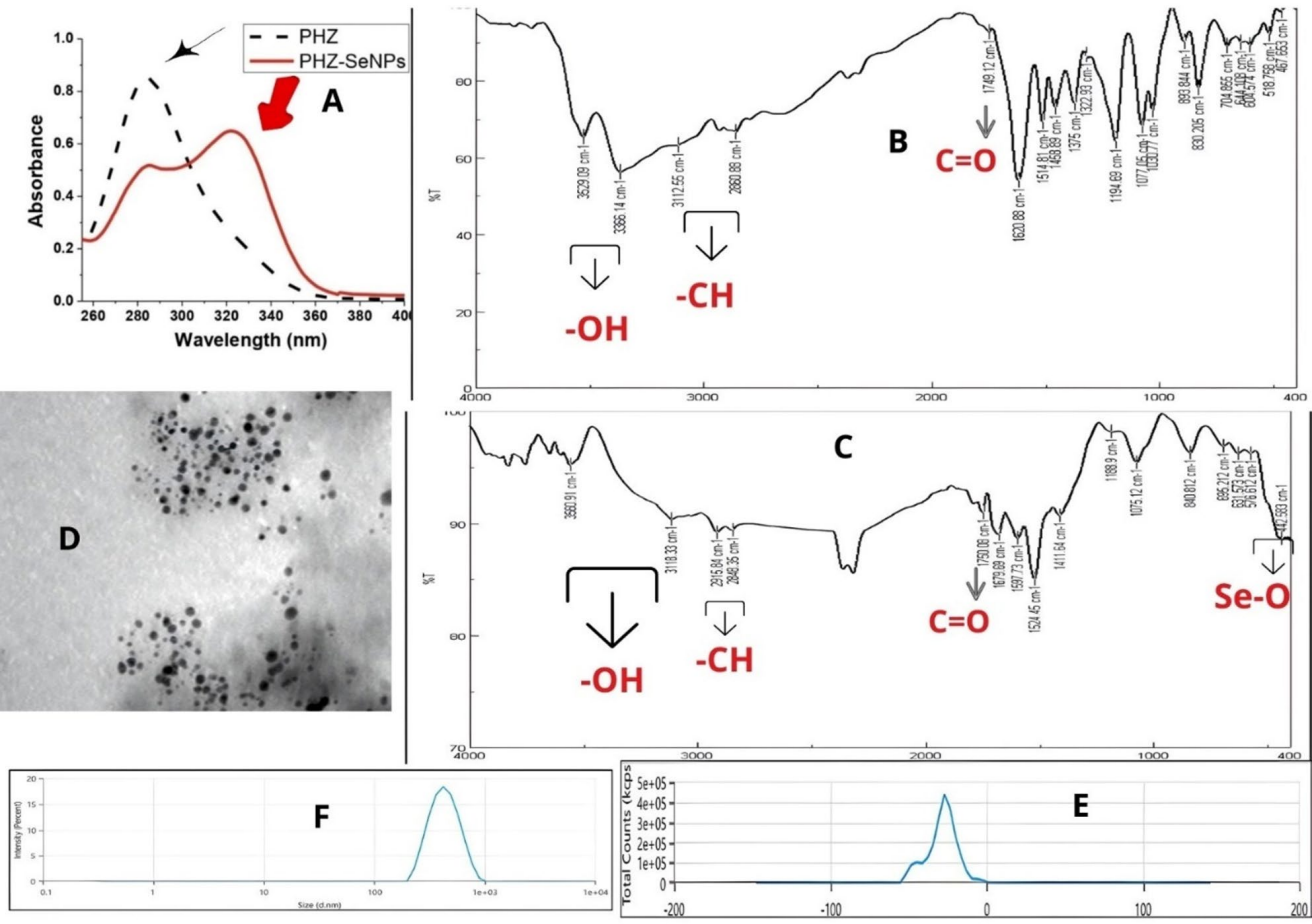
Characterization of selenium nanoparticles synthesized by PHZ; **A** The ultraviolet-visible absorption bands of PHZ & PHZ-SeNPs solution, **B** FT-IR absorption spectrum for PHZ, **C** FT-IR absorption spectrum of PHZ-mediated selenium nanoparticles (PHZ-SeNPs), **D** Transmission electron microscopy (TEM) image of the greenly prepared PHZ-SeNPs showing spherical morphology and an average size (5–11 nm), **E** Zeta potential of prepared PHZ-SeNPs (− 28.77 ± 9.5 mV), **F** Zeta average size distribution of the prepared PHZ-SeNPs (395 nm)

The optical characteristics of Se NPs were examined using a double-beam UV-Vis spectrophotometric technique. When these nanoparticles were being prepared, color changes were visually detected during its change (yellow to red color) after 5–6 h. The absorption peak of PHZ was detected at λ_max_ 284 nm. On the other hand, new absorption peak in the sample of PHZ-SeNPs which was recorded at λ_max_ 322 nm which reconfirmed formation of Se NPs using PHZ (Fig. 1A).

The participation of –OH, C = O, and C–O functional groups, linked to the bioactive molecules of PHZ coating on the outer layer of Se NPs, was verified by FT-IR spectroscopy. The wave number range of this functional group was 400 to 4000 cm^−1^ (one cm^− 1^ resolution, Fig. 1B, C). The new peak which appeared at 3560.91 cm^− 1^ and absorption bands from 800 –400 cm^− 1^ (840.812 cm^− 1^, 695.212 cm^− 1^, 631.573 cm^− 1^, 576.612 cm^− 1^ and 442.583 cm^− 1^) confirm the formation of PHZ-SeNPs.

The shape, size, distribution, and potential of PHZ-SeNPs were described using TEM and zeta studies. TEM micrograph of PHZ showed successful green synthesis of spherically shaped nanoparticles with average Se NPs size ranging from 5 to 11 nm (Fig. 1D). Zeta potential of the surface charge of the prepared materials was (− 28.77 ± 9.5 mV) as shown in Fig. 1E. The zeta average size distribution reached 395 nm (Fig. 1F). The result for poly-dispersity index (PDI) is 0.1099.

### Determination of loading capacity (LC) and encapsulation efficiency (EE)

The encapsulation efficiency (EE %) and loading capacity (LC %) of PHZ within the PHZ-SeNPs were assessed by UV–Vis spectrophotometry at λ_max_ = 284 nm. The EE% and LC% values were 38.96% and 42.8%, respectively.

### Assessments of the antioxidant’s capabilities of the PHZ and PHZ-SeNPs using DPPH radical scavenging assay

**Fig. 2:**
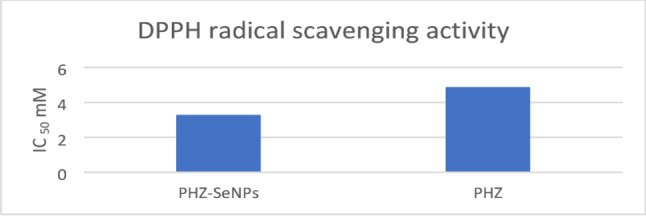
Comparison of DPPH radical scavenging activity of PHZ and PHZ-SeNPs on the bases of IC_50_ values (mM)

In Fig. 2, each column represents the concentration required to inhibit 50% of DPPH radicals (IC₅₀). PHZ-SeNPs exhibited higher antioxidant activity than free PHZ, as indicated by a lower IC₅₀ value (3.28 mM vs. 4.87 mM), demonstrating that nanoparticle formulation enhances the radical scavenging capacity of PHZ.

#### Erythrocytes glucose uptake in hyperglycemic conditions with and without the addition of PHZ and PHZ-SeNPs

**Fig. 3:**
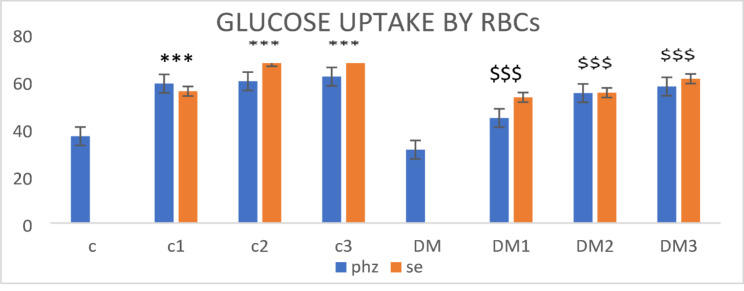
% Glucose uptake by red blood cells (RBCs) of controls and diabetic rats, with and without the addition of PHZ and PHZ-SeNPs. C: RBCs of control rats, c1: RBCs of control rats+25 µl of PHZ or PHZ-SeNPs (25 mg/dL), c2: RBCs of control rats+50 µl of PHZ or PHZ-SeNPs (50 mg/dL) c3: RBCs of control rats+100 µl of PHZ or PHZ-SeNPs (100 mg/dL), DM: RBCs of diabetic rats, DM1: RBCs of diabetic rats+25 µl of PHZ or PHZ-SeNPs (25 mg/dL), DM2: RBCs of diabetic rats+50 µl of PHZ or PHZ-SeNPs (50 mg/dL), DM3: RBCs of diabetic rats+100 µl of PHZ or PHZ-SeNPs (100 mg/dL). The significant levels when comparing the rats’ results to the healthy control were denoted by the symbols *, **, and ***. $$$,$$, $ represent the significant levels compared with the diabetic control group. ***, $$$: *p* < 0.001, **, $$: *p* < 0.01, *, $: *p* < 0.05, ns: *p* > 0.05

After incubation of erythrocytes of the healthy control and diabetic rats in hyperglycemic PBS at 37 °C for 2 h with and without addition of 25 mg/dL, 50 mg/dL and 100 mg/dL of PHZ and PHZ-SeNPs, the % of glucose uptake in erythrocytes of all diabetes-induced rats were decreased versus that of the control. Conversely, when PHZ and PHZ-SeNPs were added, the erythrocytes’ glucose uptakes were higher than when they are not (Fig. 3).

### Determination of LD_50_ of PHZ-SeNPs

After 24 h, mortality was observed. The LD_50_ of PHZ-SeNPs was determined to be 173.68 mg/kg body weight.

### Effects of PHZ, PHZ-SeNPs and insulin treatments on FBG, HbA1c, insulin levels and HOMA-IR

**Fig. 4:**
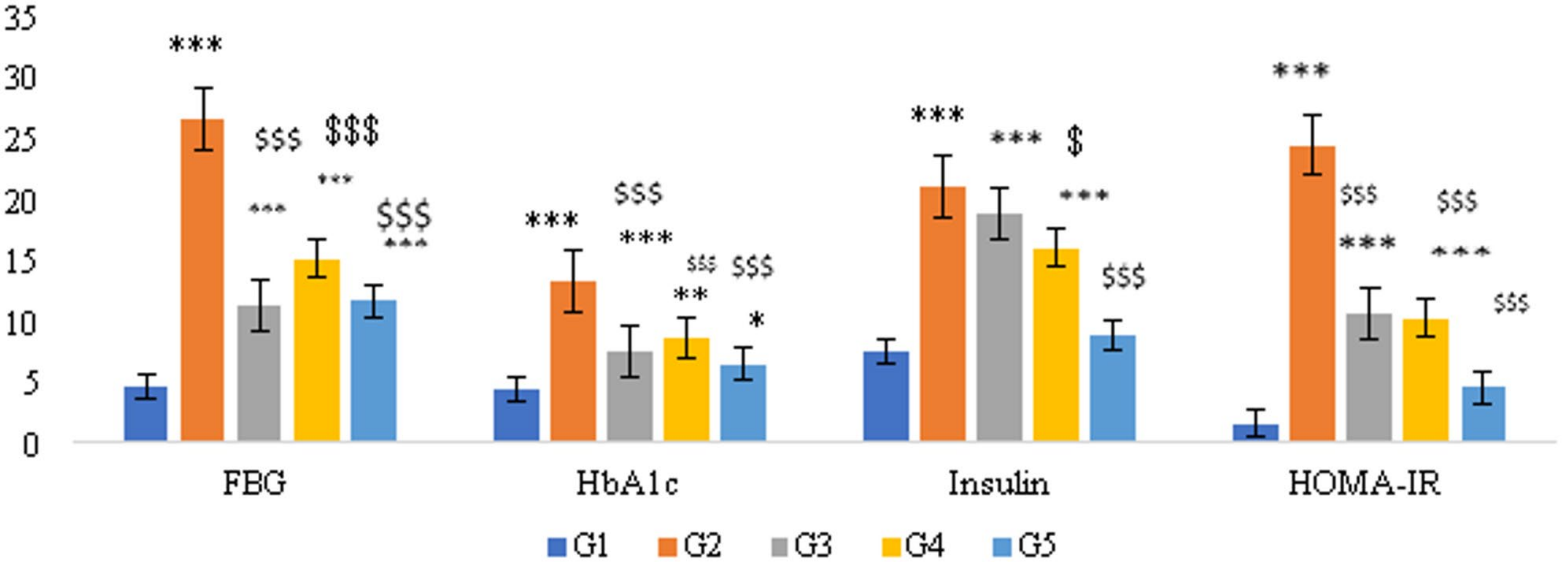
Effects of phlorizin (PHZ), phlorizin-selenium nanoparticles (PHZ-SeNPs) and insulin on FBG, HbA1c, insulin levels and HOMA-IR. G1 (Healthycontrol group), G2 (Diabetic control group), G3 (Diabetic + Sc-insulin-treated group), G4 (Diabetic+Oral Phlorizin-treated group), G5 (Diabetic + I.P PHZSeNPs-treated group). Fasting blood glucose (FBG), Glycated hemoglobin (HbA1c), calculation of homeostatic model assessment of insulin resistance(HOMA-IR). The significant levels when comparing the rats’ results to the healthy control were denoted by the symbols *, **, and ***. $, $: the significantlevels obtained by comparing the rats’ and the diabetic control group’s results. ***, $$$: *p* < 0.001, **, $$: *p* < 0.01, *, $: *p* < 0.05, ns: *p* > 0.05

Injection of STZ (i.p.) was used to induce diabetes in rats. The mean FBG values in the whole blood of diabetic rats were significantly higher compared to those of healthy rats (M ± SD= 4.52 ± 0.5 mmol/l; *p* < 0.001). The mean FBG values in PHZ- or PHZ-SeNPs- treated rats were statistically significantly lower (15 ± 3.7 mmol/l and 11.49 ± 1.93 mmol/l, respectively; *p* < 0.001) compared to those of the diabetic-untreated rats (26.54 ± 1.7 mmol/l). Similar trends were observed for insulin, HbA1c, and HOMA-IR, as illustrated in Fig. 4.

**Fig. 5:**
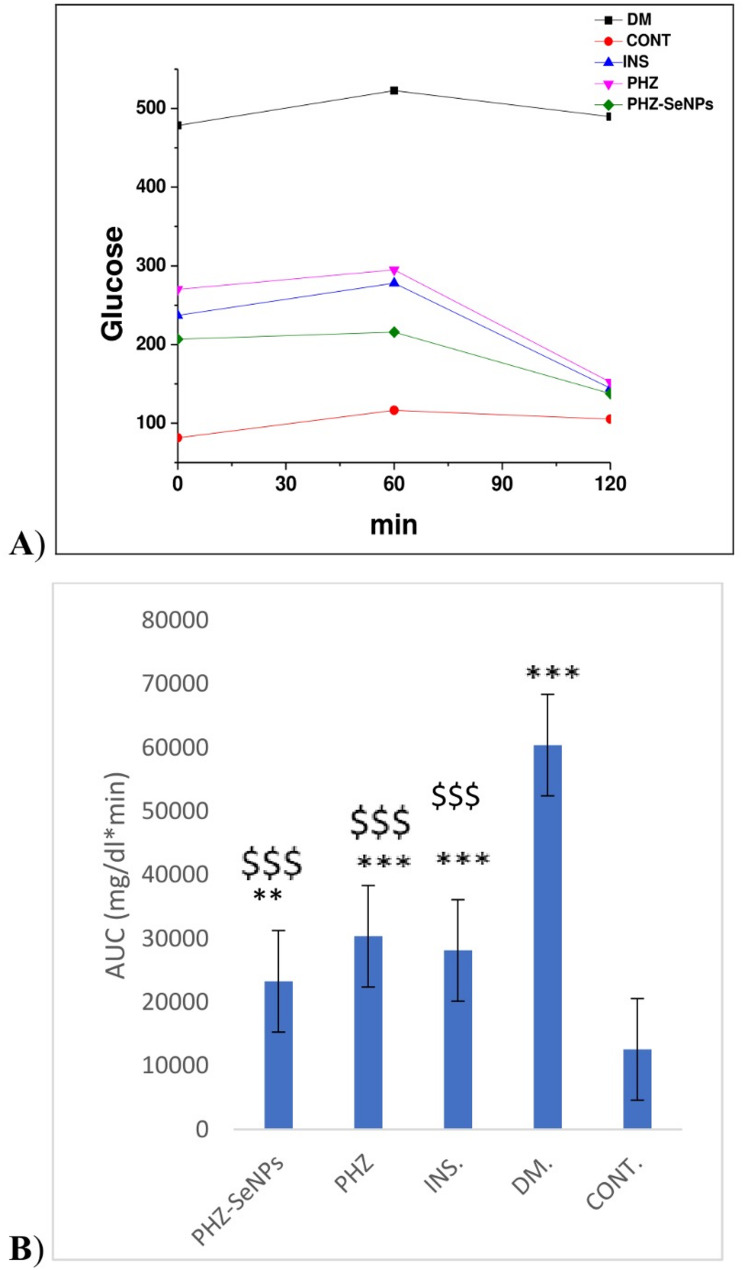
**A** Effects of phlorizin (PHZ), insulin (INS) and phlorizin-selenium nanoparticles (PHZ-SeNPs) on blood glucose tolerance curve, **B** Calculated area under curves (AUCs) in control, diabetic and treated rats. PHZ-SeNPs= Diabetic+phlorizin-selenium nanoparticles-treated group, PHZ= Diabetic+phlorizin-treated group, INS= Diabetic+insulin-treated group, DM= Diabetic control group, CONT= Healthy control group. The significant levels when comparing the rats’ results to the healthy control were denoted by the symbols *, **, and ***. The significant levels obtained by comparing the rats’ results to the diabetic control group’s results were denoted by the symbol $, $$, $$$ $$$: *p* < 0.001, **, $$: *p* < 0.01, *, $: *p* < 0.05, ns: *p* > 0.05

With respect to glucose tolerance testing, blood glucose levels were measured at 0, 60, and 120 min for all experimental groups. As expected, the mean levels of glucose were the highest at 60 min post-glucose administration. After 120 min, the mean blood glucose levels in treated rats were nearly normalized compared to the diabetic group (Fig. 5A).

The areas under curve (AUC) for each curve were computed using the data from Fig. 5A. Diabetic rats exhibited AUC considerably higher when compared with that healthy group (*p* < 0.001). Furthermore, the AUCs of the insulin-, PHZ-, and PHZ-SeNPs-treated diabetic rats were significantly lower (*p* < 0.001) compared with the untreated diabetic rats. These AUCs’ outcomes were depicted in Fig. 5B.

Table 1’s results showed an extremely significantly increase in the mean leptin level in sera of diabetic rats as well as the calculated LAR when compared to this of the health one (2.69 ± 0.5 ng/ml and 0.043 ± 0.016, respectively; *p* < 0.001). When leptin value in sera of PHZ-treated and PHZ-SeNPs rats were compared with that of diabetic-untreated rats (27.76 ± 4.15 ng/ml), the formers groups showed statistically significant decreases; especially those which were treated with the PHZ-SeNPs (12.2 ± 1.99 and 7.52 ± 2.3 ng/ml, sequentially; *p* < 0.001). However, results of ADP levels and A/L ratio of diabetic rats were extremely significantly decreased (23.4 ± 7.06 ng/ml and 0.89 ± 0.3, respectively) compared with those of healthy rats (66.84 ± 15.3 ng/ml and 26.63 ± 11.7, respectively, *p* < 0.001). Further, PHZ- or PHZ-SeNPs-treated diabetic rats showed considerably higher mean serum ADP levels and A/L ratio than the untreated diabetic rat (Table 1).

**Table 1:** Effects of phlorizin (PHZ), phlorizin-selenium nanoparticles (PHZ-SeNPs) and insulin on ADP, leptin, LAR and A/L ratio

Variables	Leptin (ng/ml)	Adiponectin (ng/ml)	LAR	A/L ratio
Healthy control group	2.69 ± 0.5	66.84 ± 15.3	0.043 ± 0.016	26.63 ± 11.7
Diabetic control group	27.76 ± 4.15^***^	23.4 ± 7.06^***^	1.33 ± 0.6^***^	0.89 ± 0.3^***^
Diabetic+Insulin-treated group	18.4 ± 5.3^***, $$^	35.8 ± 5.9^***, $$^	0.54 ± 0.2^***, $$$^	2.16 ± 0.9^***, ns^
Diabetic+Phlorizin-treated group	12.2 ± 1.99^**, $$$^	32.06 ± 3.38^***, ns^	0.39 ± 0.09^ns, $$$^	2.72 ± 0.7^***, ns^
Diabetic+PHZ-SeNPs-treated group	7. 52 ± 2.3^ns, $$$^	42.09 ± 5.9^***, $$$^	0.19 ± 0.07^ns, $$$^	6.44 ± 3.5^***, $^

The significant levels when comparing the rats’ results to the healthy control were denoted by the symbols *, **, and ***. The significant levels obtained by comparing the rats’results to the diabetic control group’s resultswere denoted by the symbols. ***,$$$: *p* < 0.001, **, $$: *p* < 0.01, *, $: *p* < 0.05, ns: *p* > 0.05

As shown in Table 2, the mean cholesterol level in the sera of the untreated diabetic rats was 343.6 ± 104.2 mg/dL. Further, the mean cholesterol levels in sera of diabetic rats treated with insulin as well as PHZ were 253.8 ± 33.7 mg/dL and 201.5 ± 3.13 mg/dL, respectively. These mean levels were significantly higher than those of the healthy rats (82.11 ± 29.3 mg/dL, *p* < 0.001). Rats with diabetes treated with PHZ-SeNPs had a mean serum total cholesterol level of 148.4 ± 38.6 mg%. This value was significantly lower than that of diabetic untreated rats, but still higher than the control group.

Concerning the mean triglyceride levels in sera of insulin-, PHZ- or PHZ-SeNPs-treated diabetic rats, all showed extremely significantly decreases (203.67 ± 41.3 mg/dL, 143.8 ± 27.5 mg/dL, 106.83 ± 23.49 mg/dL, respectively) compared with the untreated diabetic rats (376.4 ± 29.4 mg/dL, *p* < 0.001); particularly those which were treated with PHZ-SeNPs.

**Table 2:** Effects on lipid profiles of insulin, phlorizin (PHZ), phlorizin-selenium nanoparticles (PHZ-SeNPs)

Variables	Healthy control group	Diabetic control group	Diabetic+Insulin-treated group	Diabetic+Phlorizin-treated group	Diabetic+PHZ-SeNPs-treated group
T.C. (mg/dL*)*	82.11 ± 29.3	343.6 ± 104.2^***^	253.8 ± 33.7^***,^^$$^	201.5 ± 3.13^***,^^$$$^	148.4 ± 38.6^*,^^$$$^
T.G. *(*mg/dL*)*	74 ± 15.7	376.4 ± 29.4^***^	203.67 ± 41.3^***,^^$$$^	143.8 ± 27.5^**,^^$$$^	106.83 ± 23.49^*,^^$$$^
HDL *(*mg/dL*)*	41.63 ± 8.49	22.15 ± 1.75^***^	29.57 ± 6.87^***,^^$^	32.95 ± 3.93^**,^^$$$^	36.67 ± 8.59^*,^^$$$^
LDL *(*mg/dL*)*	25.68 ± 18.7	249.5 ± 109.7^***^	183.5 ± 29.4^***,^^$^	139.7 ± 6.4^***,^^$$$^	93.3 ± 33.17^*,^^$$$^
VLDL *(*mg/dL*)*	14.8 ± 3.15	71.9 ± 8.16^***^	40.73 ± 8.26^***,^^$$$^	28.77 ± 5.49^**,^^$$$^	18.37 ± 9.3^*,^^$$$^
T.lipid*(*mg/dL)	382.69 ± 66.6	1229.39 ± 392.6^***^	829.95 ± 87.7^***,^^$$$^	785.7 ± 164.4^***,^^$$$^	510.18 ± 109.9^*,^^$$$^

-The significant levels when comparing the rats’ results to the healthy control were denoted by the symbols *, **, and ***.The significant levels obtained by comparing the rats’results to the diabetic control group’s results were denoted by symbols : *p* < 0.001, **, $$: *p* < 0.01, *, $: *p* < 0.05, ns: *p* > 0.05

#### Impact of PHZ, PHZ-SeNPs, and insulin treatments some liver-related markers in diabetic rats

The mean ALT and AST activities in diabetes-induced rats were highly significant elevation but the mean levels of albumin and total proteins were extremely significant decrease compared with healthy rats (*p* < 0.001). However, the mean ALT and AST activities in diabetic rats which were treated with PHZ-SeNPs were extremely significantly decreased but the mean levels of albumin and total proteins were highly significant increase compared with the diabetic groups (Table 3).

The mean uric acid, urea, and creatinine levels in sera of diabetes-induced rats were considerably elevated (*p* < 0.001) compared with healthy rats. On contrary, the mean values of these 3 functions in sera of diabetic rats which were treated with PHZ-SeNPs were significantly lower compared with diabetic rats (Table 3).

**Table 3:** Liver and kidney functions tests in sera of control rats and in sera of those with diabetes and diabetic-treated rat’s groups

Variables	Healthy control group	Diabetic control group	Diabetic+Insulin-treated group	Diabetic+Phlorizin-treated group	Diabetic+PHZ-SeNPs-treated group
Liver functions tests					
ALT (U/ml)	33.31 ± 11.65	111.74 ± 16.2^***^	38.85 ± 17.9^ns, $$$^	48.2 ± 18.56^ns, $$$^	35.26 ± 10.7^ns, $$$^
*AST(U/ml)*	*43.43 ± 17.35*	*133.68 ± 30.1* ^***^	*52.6 ± 10.38* ^*ns, $$$*^	*59.18 ± 14.8* ^*ns, $$$*^	*52.18 ± 20.36* ^*ns, $$$*^
*Albumin(g/dL)*	*3.02 ± 0.36*	*2.19 ± 0.36* ^***^	*2.82 ± 0.15* ^*ns, $$$*^	*2.6 ± 0.2* ^*, $^	*2.84 ± 0.23* ^*ns, $$$*^
*T.Proteins(g/dL)*	*6.2 ± 0.89*	3.16 ± 1.66***	*5.98 ± 1.04* ^*ns, $$$*^	*5.84 ± 1.08* ^*ns, $$$*^	*6.1 ± 0.7* ^*ns, $$$*^
*Kidneys functions tests*					
*Creatinine* *(mg/dL)*	*0.39 ± 0.2*	*3.57 ± 1.28* ^***^	*0.51 ± 0.24* ^*ns, $$$*^	*1.67 ± 0.66* ^*, $$$^	*0.85 ± 0.29* ^*ns, $$$*^
*Uric acid* *(mg/dL)*	*2.99 ± 0.32*	*9.88 ± 1.5* ^***^	*3.38 ± 0.22* ^*ns, $$$*^	*6.01 ± 0.59* ^***, $$$^	*4.14 ± 0.58* ^*, $$$^
*Urea* *(mg/dL)*	*31.78 ± 5.8*	*189.6 ± 39.7* ^***^	*79*,6 ± 8.5^***, $$$^	*123.2 ± 7.3* ^*** $$$^	*101.89 ± 13.2* ^***, $$$^

values were expressed as mean±standard deviations -The significant levels when comparing the rats’ results to the healthy control were denoted by the symbols *, **, and ***.The significant levels obtained by comparing the rats’results to the diabetic control group’s results were denoted by symbols : *p* < 0.001, **, $$: *p* < 0.01, *, $: *p* < 0.05, ns: *p* > 0.05

The mean level of MDA in red blood cells of diabetic rats treated with PHZ-SeNPs was significantly decreased (0.099 ± 0.011 mmol/g, *p* < 0.001) compared to diabetic untreated rats (0.287 ± 0.103 mmol/g). Similarly, the mean serum NO level was significantly reduced in PHZ-SeNPs–treated diabetic rats (2.77 ± 0.75 µmol/l, *p* < 0.001) compared to diabetic controls (6.98 ± 1.25 µmol/l). NO mean serum level of diabetic rats which treated with PHZ-SeNPs were highly significant decrease compared with diabetic rats (0.287 ± 0.103 mmol/g versus 6.98 ± 1.25 µmol/l, respectively, *p* < 0.001). conversely, the mean CAT activity and TAC in diabetic groups were somewhat lower (*p* < 0.001) if compared with the untreated diabetic group ( 0.031 ± 0.013 U/g protein and 7.42 ± 1.05 mmol/l, respectively). In addition, as indicated in Table 4, the mean GSH level and SOD activity in diabetes-induced rats which treated with PHZ-SeNPs were highly significant increase (*p* < 0.001) compared with the diabetic rats’ values.

**Table 4:** The average levels of oxidative stress markers and antioxidants assayed for the healthy control rats versus those of diabetic-non treated and diabetic-treated groups

Variables	MDA (mmol/g)	NO (µmol/l)	GSH (mmol/g)	CAT (U/g protein)	TAC (mmol/l)	SOD (%inhibition)
Healthy control group	0.085 ± 0.014	2.79 ± 1.19	5.09 ± 0.83	0.129 ± 0.03	13.03 ± 0.85	43.03 ± 4.59
Diabetic control group	0.287 ± 0.103^**^	6.98 ± 1.25^**^	2.16 ± 0.63^**^	0.031 ± 0.013^***^	7.42 ± 1.05^***^	19.2 ± 2^***^
Diabetic+Insulin-treated group	0.230 ± 0.062^**,^^ns^	5.88 ± 0.53^**,^^$^	2.84 ± 0.34^**,^^$^	0.07 ± 0.02^***,^^$$$^	10.13 ± 0.47^***,^^$$$^	30.48 ± 3.44^***,^^$$$^
Diabetic+Phlorizin-treated group	0.192 ± 0.086^**,^^$$^	4.51 ± 0.66^**,^^$$$^	3.37 ± 0.42^**,^^$$$^	0.081 ± 0.013^***,^^$$$^	11.86 ± 0.65^*,^^$$$^	38.42 ± 3.72^*,^^$$$^
Diabetic+PHZ-SeNPs-treated group	0.099 ± 0.011^*,^^$$^	2.77 ± 0.75^ns,^^$$^	4.03 ± 0.7^**,^^$$$^	0.098 ± 0.013^**,^^$$$^	12.82 ± 0.56^ns,^^$$$^	41.13 ± 4.23^ns,^^$$$^

values were expressed as mean±standard deviations -The significant levels when comparing the rats’ results to the healthy control were denoted by the symbols *, **, and ***.The significant levels obtained by comparing the rats’results to the diabetic control group’s results were denoted by symbols . ***, $$$: *p* < 0.001, **, $$: *p* < 0.01, *, $: *p* < 0.05, ns: *p* > 0.05

#### Liver glycogen

**Fig. 6:**
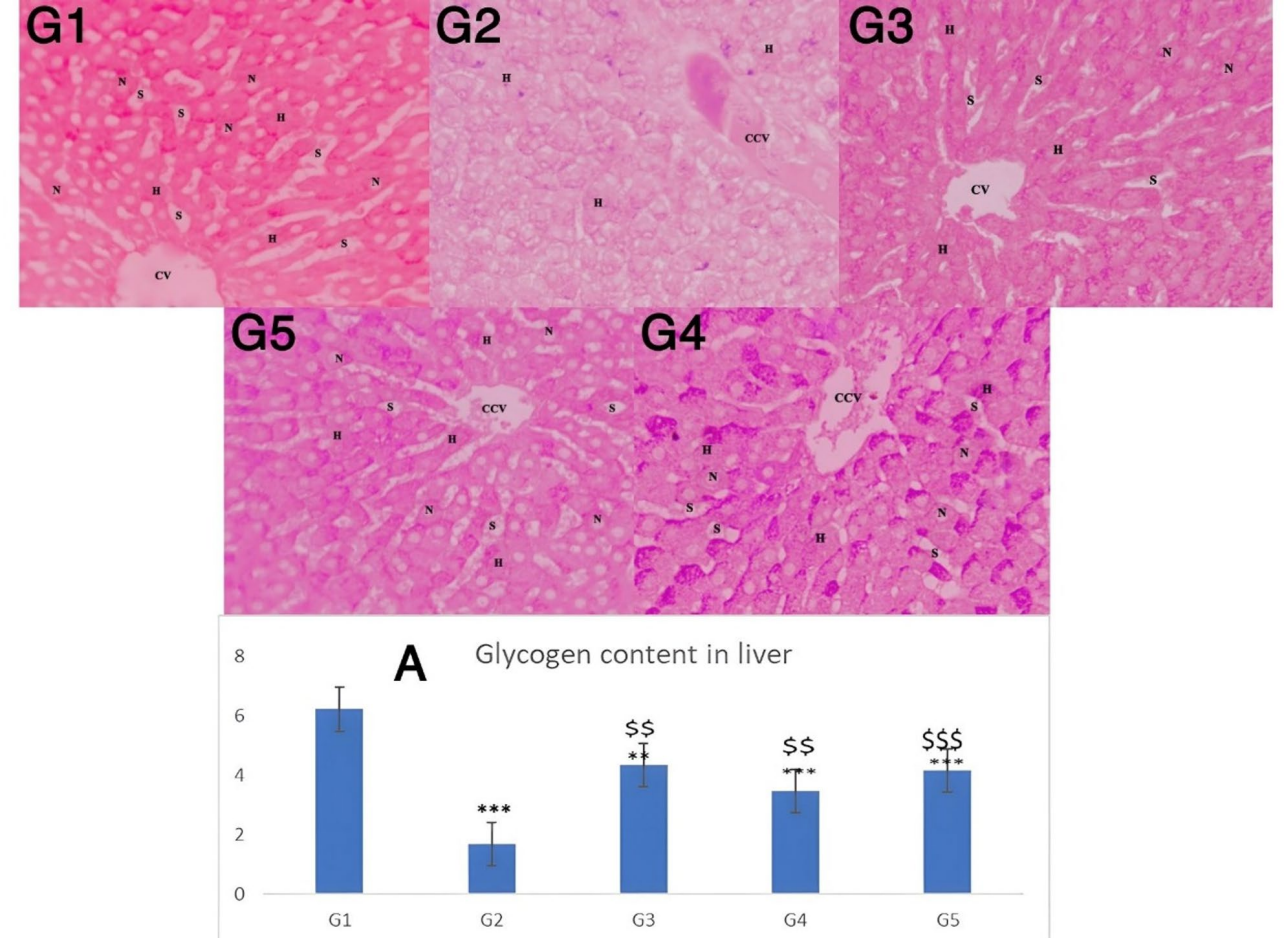
Histopathology of liver sections stained with PAS and its summation **A**. ***G1***: Photomicrograph of healthy control group. **G2**: Photomicrograph of diabetic control group. **G3**: Photomicrograph of diabetic+insulin-treated group. **G4**: Photomicrograph of diabetic+PHZ-treated group. **G5**: Photomicrograph of diabetic+PHZ-SeNPs treated group. H: hepatocytes, N: nucleus and S: sinusoid (PAS, 400X). The significant levels when comparing the rats’ results to the healthy control were denoted by the symbols *, **, and ***. $, $$, $$$ represent the significant levels compared with the diabetic control group. ***, $$$: *p* < 0.001, **, $$: *p* < 0.01, *, $: *p* < 0.05, ns: *p* > 0.05

Figure 6, illustrates the histopathology of liver sections stained with PAS among different experimental groups. In the control group (Fig. 6G1) showed uniform distribution of PAS positivity in the cytoplasm of hepatocytes surrounding the central vein (CV), sinusoid (S), nucleus (N) and hepatocytes (H) (PAS, 400X). In contrast, in diabetic group (Fig. 6G2) the staining showed diminished PAS positivity reaction in the cytoplasm of hepatocytes surrounding CV, congested central vein (CCV) and H (PAS, 400X).The insulin-treated group (Fig. 6G3) demonstrated restoration of the glycogen distribution in the hepatocytes radiating from CV, H, N and S (PAS, 400X). Similarly, in the PHZ-treated group (Fig. 6G4), the glycogen distribution was restored in the hepatocytes radiating from CV, CCV. Notably, the PHZ-SeNPs treated group (Fig. 6G5) exhibited more restoration of the glycogen distribution in the hepatocytes radiating from CV, CCV, H, N and S (PAS, 400X). The mean glycogen levels were calculated from PAS staining and the results were depicted in Fig. 6A. In this regard it was observed that the hepatic tissue’s glycogen contents were significantly lower (*p* < 0.001) in rats with STZ-induced diabetes versus that of the untreated control (6.23 ± 1.6 mg/g tissue). Following PHZ-SeNPs treatment, the diabetes-induced rats noted a remarkable increase in their liver glycogen contents if compared with that of the diabetic control (1.7 ± 0.46 mg/g tissue).

### Histopathological changes in liver, kidneys and pancreatic tissues

**Fig. 7:**
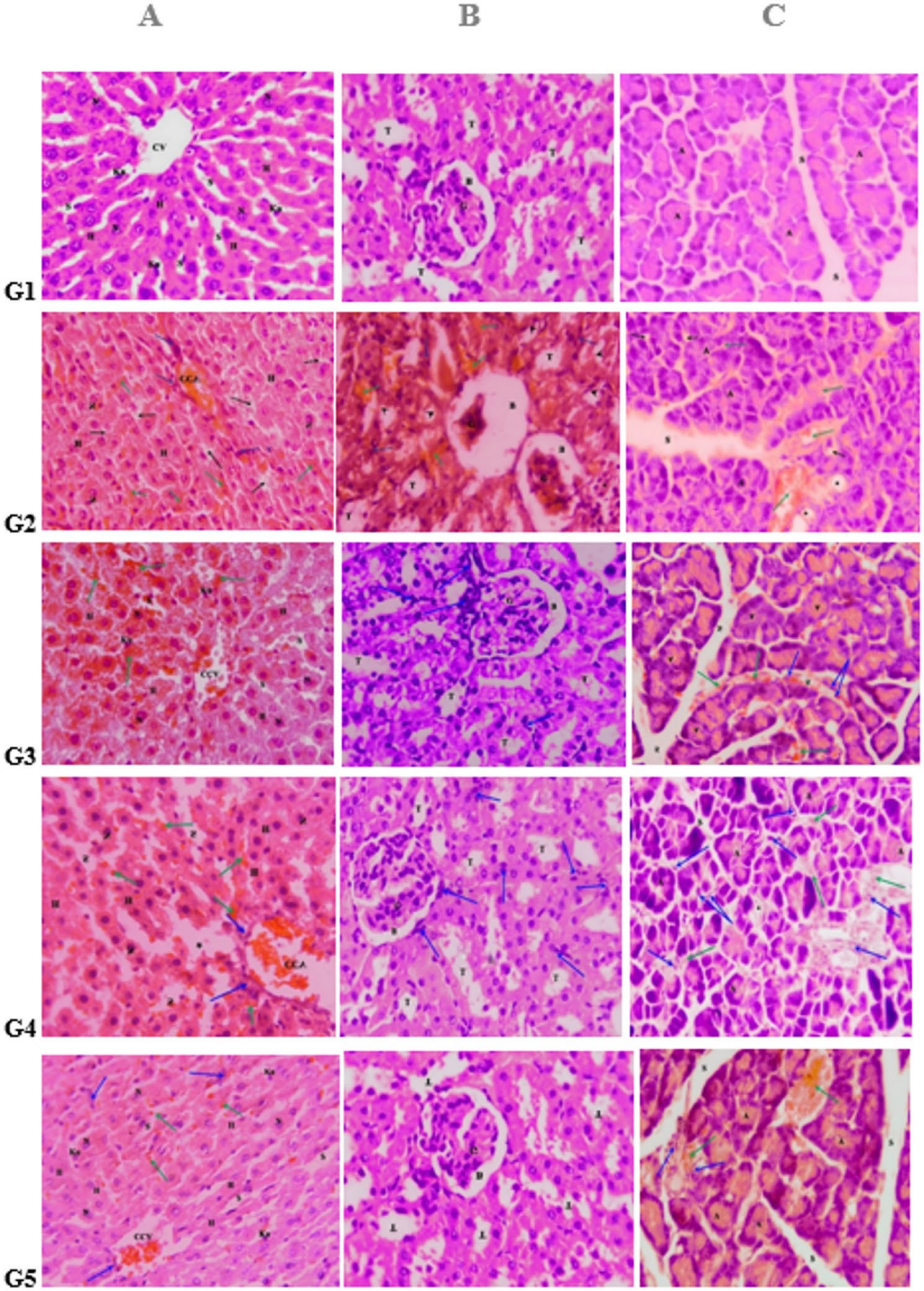
Histopathological findings of liver **A**, histopathological findings of kidney **B** and histopathology of pancreas **C**: (G1) Photomicrograph of heathy control group. (G2) Photomicrograph of diabetic control group. (G3) Photomicrograph of diabetic+insulin-treated group. (G4) Photomicrograph of diabetic+PHZ treated group. (G5) Photomicrograph of diabetic+PHZ-SeNPs-treated group. S indicates widened connective tissue septae, necrosis*, hemorrhage (green arrow) and vacuolated acini (black arrow). pancreatic acini (**A**), thin connective tissue septae (S), and inflammatory cells infiltration (blue arrow)

Histopathological evaluation of liver and kidney tissues was performed qualitatively, describing lesion severity as mild, moderate, or severe. Figure 7A illustrates the histopathological changes in liver sections among different experimental groups. In the healthy control group (Fig. 7G1), the liver exhibited normal hepatic architecture characterized by a clearly defined central vein, well-organized hepatocytes with distinct nuclei, intact blood sinusoids, and normally distributed Kupffer cells. In contrast, the diabetic control group (Fig. 7G2) revealed severe hepatic damage, including a congested central vein, loss of hepatocytes boundaries, vacuolated hepatocytes, blood hemorrhage between hepatocytes and inflammatory cells infiltration. The insulin-treated group (Fig. 7G3) demonstrated moderate restoration of hepatic architecture with congested central vein, hepatocytes and distinct nuclei comparable to those of normal, although severe hemorrhage between hepatocytes and within sinusoids was still evident. Similarly, the PHZ-treated group (Fig. 7G4) showed mild to moderate improvement towards normal architecture with the presence of congested central vein, hepatocytes, sinusoids, and nuclei. However, hemorrhage and inflammatory cell infiltration persisted. Notably, the PHZ-SeNPs–treated group (Fig. 7G5) exhibited a more pronounced restoration of normal hepatic architecture, with well-defined central vein, hepatocytes, sinusoids, and nuclei. However, only mild hemorrhage and minimal inflammatory cells infiltrations were observed when compared to the other treatments groups.

Figure 7B illustrates the histopathological finding of kidney sections among different experimental groups. The diabetic control group of rats revealed severe renal damage, including glomerular atrophy, widened Bowmen’s space and degenerated renal tubules. extensive interstitial hemorrhage, inflammatory cell infiltration, and focal necrosis (Fig. 7G1). In contrast, in the healthy control group, the kidney exhibited normal renal architecture which was characterized by normal glomeruli, normal Bowmen’s space, and intact renal tubules (Fig. 7G2). Insulin-treated group (Fig. 7G3) demonstrated moderate restoration, with normal glomeruli, normal Bowmen’s space, and normal renal tubules; however, moderate interstitial and periglomerular inflammatory infiltration persisted. Similarly, PHZ-treated group (Fig. 7G4) showed mild to moderate improvement, with slightly atrophied glomeruli, partially restored tubules, and moderate inflammatory cell infiltration. Notably, the PHZ-SeNPs-treated group exhibited marked restoration with mild residual lesions, showing normal glomeruli and normal renal tubules and only minimal inflammatory cell infiltration (Fig. 7G5).

In Fig. 7C, the control displayed normal pancreatic architecture with well-organized pancreatic acini and thin connective tissue septae (Fig. 7G1). In contrast, the diabetic control group (Fig. 7G2) exhibited severe pancreatic damage, characterized by disorganized architecture, hemorrhage, and dense inflammatory cell infiltration. Treatment with insulin (Fig. 7G3) markedly restored the normal pancreatic structure, with preservation of acini and septae and a reduction in pathological alterations. Similarly, the PHZ-treated group (Fig. 7G4) showed partial restoration of pancreatic architecture; however, hemorrhage and severe inflammatory infiltration were still evident. Surprisingly, the PHZ-SeNPs–treated group (Fig. 7G5) demonstrated substantial restoration of normal pancreatic architecture, with clearly defined acini and thin connective tissue septae approaching that of the control group.

Table S1 and Figure S1 present the semi-quantitative analysis, showing approximately four islets per 10 HPFs in the control group, with 5–10 cells per islet. In the diabetic (DM) group, pancreatic sections demonstrated an increased number and size of β-cell islets compared with the control group, with approximately five islets per 10 HPFs and 50–100 cells per islet, suggestive of a compensatory β-cell response during early type II diabetes mellitus. In insulin-treated, PHZ, and nano-treated groups, pancreatic tissue showed few β-cell islets, with one focus per 10 HPFs and a reduced number of cells per islet (See supplement file).

As presented in Table 5, HOMA-IR showed a significant positive correlation with FBG, insulin, HbA1c, LAR, T.C., T.G., LDL, VLDL, total lipids, and leptin levels, while exhibiting a negative correlation with HDL, ADP, and the A/L ratio. Moreover, ADP and A/L ratio were negatively correlated with FBG, INS., HbA1c, T.G., LDL, VLDL, and total lipids, whereas leptin was negatively correlated with HDL. A strong positive association was also observed between HDL, adiponectin, and the A/L ratio.

**Table 5:** Values of the correlation’s coefficients between the individual values of FBG, HbA1c, insulin, HOMA-IR, ADP, leptin, A/L ratio and LAR with each other’s and with lipids profile

Variables	FBG (mg/dL)	HbA1c	Insulin (µIU/m)	HOMA-IR	ADP (ng/ml)	Leptin (ng/ml)	A/L	LAR
HbA1c *r* *P*	0.98 *p* < 0.001	1	0.391 *p* < 0.05	0.80 *p* < 0.001	− 0.65 *p* < 0.001	0.69 *p* < 0.001	-0.56 *p* < 0.001	0.61 *p* < 0.001
FBG *r* *P*	1	0.98 *p* < 0.001	0.48 *p* < 0.01	0.85 *p* < 0.001	-0.71 *p* < 0.001	0.72 *p* < 0.001	-0.55 *p* < 0.001	0.67 *p* < 0.001
Insulin r P	0.48 *p* < 0.01	0.391 *p* < 0.05	1	0.80 *p* < 0.001	-0.54 *p* < 0.001	0.65 *p* < 0.001	-0.47 *P* < 0.01	0.53 *p* < 0.001
HOMA-IR r P	0.85 *p* < 0.001	0.80 *p* < 0.001	0.80 *p* < 0.001	1	-0.59 *p* < 0.001	0.77 *p* < 0.001	-0.45 *p* < 0.01	0.68 *p* < 0.001
ADP r P	-0.71 *p* < 0.001	− 0.65 *p* < 0.001	-0.54 *p* < 0.001	-0.59 *p* < 0.001	1	-0.60 *p* < 0.001	0.89 *p* < 0.001	-0.68 *p* < 0.001
Leptin r P	0.72 *p* < 0.001	0.69 *p* < 0.001	0.65 *p* < 0.001	0.77 *p* < 0.001	-0.60 *p* < 0.001	1	-0.46 *p* < 0.01	0.73 *p* < 0.001
A/L r P	-0.55 *p* < 0.001	-0.56 *p* < 0.001	-0.47 *p* < 0.01	-0.45 *p* < 0.01	0.89 *p* < 0.001	-0.46 *p* < 0.01	1	-0.48 *p* < 0.01
LAR r P	0.67 *p* < 0.001	0.61 *p* < 0.001	0.53 *p* < 0.001	0.68 *p* < 0.001	-0.68 *p* < 0.001	0.73 *p* < 0.001	-0.48 *p* < 0.01	1
Cholesterol r P	0.79 *p* < 0.001	0.741 *p* < 0.001	0.72 *p* < 0.001	0.798 *p* < 0.001	0.72 *p* < 0.001	0.77 *p* < 0.001	-0.6 *p* < 0.001	0.667 *p* < 0.001
Triglycerides r P	-0.79 *p* < 0.001	0.721 *p* < 0.001	0.68 *p* < 0.001	0.85 *p* < 0.001	-0.57 *p* < 0.001	0.71 *p* < 0.001	-0.41 *p* < 0.01	0.7767 *p* < 0.001
HDL r P	-0.65 *p* < 0.001	-0.621 *p* < 0.001	-0.35 *p* < 0.001	-0.58 *p* < 0.01	0.26 *p* < 0.01	-0.596 *p* < 0.001	0.16 *p* < 0.001	-0.476 *P* < 0.01
LDL r P	0.77 *p* < 0.001	0.723 *p* < 0.001	0.65 *p* < 0.001	0.74 *p* < 0.001	-0.69 *p* < 0.01	0.75 *p* < 0.001	-0.59 *p* < 0.001	0.603 *p* < 0.001
VLDL r P	0.755 *p* < 0.001	0.693 *p* < 0.001	0.69 *p* < 0.01	0.704 *p* < 0.001	-0.56 *p* < 0.001	0.704 *p* < 0.001	-0.395 *p* < 0.012	0.761 *p* < 0.001
Total lipids r P	0.69 *p* < 0.001	0.638 *p* < 0.001	0.67 *p* < 0.001	0.801 *p* < 0.001	-0.54 *p* < 0.001	0.62 *p* < 0.001	-0.399 *p* < 0.011	0.668 *p* < 0.001

-r=Pearson correlation coefficient -P= Probability

## Discussion

PHZ is a natural polyphenol glucoside, rapidly hydrolyzed into phloretin (PHT) by intestinal enzymes (lactase-phlorizin hydrolase and β-glucosidase). PHT undergoes extensive phase I and II metabolism, forming glucuronidated/sulfated conjugates that are excreted into bile, hydrolyzed back to aglycone, and reabsorbed via enterohepatic circulation. This result in poor oral bioavailability, limiting its clinical efficacy (Wang et al. 2019). In this study, Se NPs were greenly synthesized. Additionally, PHZ-SeNPs were administered intraperitoneally (i.p.) to ensure accurate systemic delivery and allows rapid and efficient bioavailability and avoid enzymatic degradation in the gut (Al Shoyaib et al. 2020).

In Fig. 1A, the UV–Vis spectrum of PHZ-SeNPs showed two characteristic absorption peaks. The first peak at 284 nm corresponds to the intrinsic absorption of PHZ, but with a markedly reduced intensity compared with free PHZ, indicating successful adsorption/loading onto the nanoparticle surface. A second peak appeared at 322 nm, which is attributed to the surface plasmon resonance (SPR)-like electron oscillation characteristic of selenium nanoparticles, confirming the formation of Se NPs (Faramarzi et al. 2020). Dual functionality of PHZ as both a natural stabilizer and a reducing agent was demonstrated. Similarly, Hadi and Mohamed (2025) synthesized Se NPs-based orange peels with a flavonoid in nature as that of PHZ, the absorption band was at 289 nm. Further, Azeem et al. (2023) synthesize Se NPs from dried peel extract of Citrus paradise with λ_max_ at 340 nm and Alam et al. (2019) synthesized Se NPs at λ_max_ of 381 nm. In our opinion, the changes in absorption maxima towards longer wavelengths may be due the increases in particle size. Generally, the stability of Se NPs was correlated with agglomeration and aggregation.

FT-IR analysis of PHZ-SeNPs revealed characteristic peaks confirming the involvement of PHZ functional groups in nanoparticle formation. The broad –OH band stretching vibrations of phenolic and hydroxyl groups, shifted from 3529.09 cm⁻¹ and 3366.14 cm⁻¹ in pure PHZ to 3560.91 cm⁻¹ and 3118.33 cm⁻¹ in the nanoparticles, which can donate electrons to reduce Se⁴⁺ ions to elemental Se⁰ (El-Zahed et al. 2021). The band observed around 1750.08 cm⁻¹ represents C=O stretching of carbonyl groups in polyphenols which was slightly shifted from 1749.12 cm ^-1^ in pure PHZ, involved in both reduction and stabilization of the nanoparticles. The bands of aromatic C=C stretching at 1679.69 cm^− 1^ and at 1597.73 cm^− 1^ also shifted, suggesting partial participation in reduction and strong interaction with the nanoparticle surface (Alam et al. 2019). The peaks at 2915.84 cm⁻¹ and 2848.35 cm⁻¹ correspond to C–H stretching in aliphatic structures as well as peaks at 1188.9 cm⁻¹ and 1075.12 cm⁻¹ correspond to C–O and C–O–C vibrations, confirming the presence of alcohols and ethers. These functional groups became more pronounced or slightly shifted, reflecting their role as capping agents, stabilizing the PHZ-SeNPs. A new peak at 442 cm⁻¹ appeared in PHZ-SeNPs, characteristic of Se⁰ lattice vibrations, confirming successful nanoparticle formation. Additional peaks at 576.61 and 631.57 cm⁻¹ suggest selenium–oxygen bonds (Se–O interactions), likely due to coordination with PHZ functional groups, supporting capping and colloidal stability (Gold et al. 2018). Thus, it was demonstrated that the PHZ use their potent repulsive forces to stabilize and cap the surface of Se NPs preventing both agglomeration as well as their aggressive aggregation (Mohamed and El‑Zahed 2024) as shown in Fig. 1B, C. In the present study PHZ-SeNPs was found to have a negative charge (− 28.77 ± 9.5 mV) which acts as a repulsive force between Se NPs grains, thus, improve their biological applications; including safe target cell delivery. Taken together, this capping property with PHZ not only provides Se NPs with flavonoids and phenolics but also stabilized the prepared PHZ-SeNPs. This stabilization prolongs the survival time of these particles and hence, prolongation of the time of sustained release of their antioxidants.

In the current study, the TEM micrograph of Se NPs confirms the successful green synthesis of spherically shaped nanoparticles with average size ranging from 5 to 11 nm (Fig. 1D). The findings of the current investigation are related to those of Alqahtani et al. (2025) who prepared spherical-shaped Se NPs but with an average size of 30 nm. Also, Azeem et al. (2023) who synthesized Se NPs from Citrus paradise dried peels extract with a nanoscale size of 45–70 nm and with rounded spherical shape. In addition, Alam et al. (2019) revealed that their spherical Se NPs sizes were in the range of 8–20 nm. In this study we prepared stronger Se NPs with an average potential of -28.77 ± 9.5 Mv (Fig. 1E), zeta distribution has homogenous particles with average size of 395 nm (Fig. 1F). These add more to the efficiency and effectiveness of increment in their surface area. The current study’s findings are almost identical to those of Ullah et al. (2021) investigation, which produced Se NPs and showed that the size distribution of Se NPs was 530 nm and their potential was at -26.9 mV. Also, in the study of Tugarova et al. (2018) the particles had − 18.5 mV. In contrast, Alam et al. (2019) reported that, their zeta potentials, which were at + 24.75, were used to determine the stability of colloidal solution. Taking together, in our study we obtained the smallest spherical size of Se NPs. These help them to reach their targets quickly and efficiently without aggregation or blood clotting. The variation in size between zeta size and TEM results may be due to the successful combination between PHZ and SeNPs.

In general, when the solute disperses in a solvent with a small poly dispersity index (PDI) and Zeta average size (Zavg), it denotes to the good dispersion of the solute in the solvent. The size distribution range of PHZ-SeNPs is highly homogenous as confirmed by measuring nanocolloidal solution PDI, which value was in the size distribution homogenous range (0–1). Studies have shown that the size, morphology, stability, and properties of the nanomaterials are influenced by the experimental conditions, the kinetics of interaction of ions with reducing agents, and adsorption processes of a stabilizing agent on the nanomaterials surface (Danaei et al. 2018; Narayanan et al. 2020). In this study, the recorded results showed that the PHZ-SeNPs formed a uniform dispersion due to the PDI was 0.1099 and Zeta avg. sizes was 395 nm. In addition, the high negative potential of the PHZ-SeNPs (-28.77 ± 9.5 mV) confirmed the high dispersity, good colloidal nature, and long-term stability without any aggregation. Nanoparticles derive their power from a high surface-area-to-volume ratio. Aggregation (irreversible clumping) and agglomeration (reversible clumping) effectively increase the particle size, drastically reducing the active surface area available for interacting with cell membranes. Small, individual particles can penetrate cell walls and membranes more easily. Clumped particles may become too large for efficient cellular uptake, reducing the antidiabetic effectiveness (El-Zahed et al. 2021). Controlling these factors ensures that the nanoparticles remain suspended in liquid media (like blood or growth broth) rather than precipitating out, which is vital for consistent dosing and clinical application. PHZ molecules act as capping agents by adsorbing onto the surface of the Se NPs nuclei during the growth phase. Their bulky molecular structures create a physical barrier (steric hindrance) that prevents the Se NPs cores from coming into direct contact and fusing. These compounds often impart a surface charge (as seen in Zeta Potential results). This creates a “repulsive shield” where nanoparticles of the same charge push away from each other, maintaining a stable, mono-dispersed suspension (Fayed et al. 2025). By binding to specific spherical faces of the Se NPs, PHZ limits the growth rate in certain directions, helping to achieve the specific shape and size (5–11 nm) reported in the current study.

In this study, we obtained red colored PHZ-SeNPs after 5–6 h. similar to those of Hadi and Mohamed (2025) who obtained Se NPs after continuous agitation for at least five hours at 70 °C ± 5, not just until the extract’s color changed, but also until the ruby-red hue persisted. Taken together, the current work disagrees with those of Alam et al. (2019) who prepared them after 3 h. We believe that this difference may be due to the difference in the concentration of the precursors and/or temperature changes.

In our study, the moderate EE% reflects the partial incorporation of PHZ during nanoparticle formation, whereas the relatively high LC% indicates a strong surface association of PHZ with selenium nanoparticles. These findings confirm that PHZ molecules were successfully adsorbed and coated onto the SeNP surface, ensuring both efficient entrapment and stable drug–nanoparticle interaction. During the green synthesis process, part of the PHZ acts as a reducing and stabilizing agent, leading to a proportion of drug molecules being weakly bound to or coating the nanoparticle surface instead of being fully encapsulated. Consequently, although the total drug association (LC%) is relatively high, the encapsulation efficiency (EE%) reflects only the fraction of PHZ strongly retained within the nanoparticle structure. This observation is consistent with previous reports on plant-mediated Se NPs exhibiting surface-bound rather than core-loaded drug configurations, which still contribute effectively to biological activity and stability (Hassan et al. 2019).

In the present study, the activities of PHZ-SeNPs’ to scavenge DPPH free radical was assessed by using DPPH. Therefore, by eliminating these free radicals after PHZ-Se NPs addition, peroxidation was stopped (Zhang et al. 2023). In this regard, the IC_50_ value of PHZ-SeNPs was lower than that of free PHZ (Fig. 6). This could be due to fact that the man-sized Se particles provided a larger surface area for chemical quenching and shielded the PHZ from the outside world.

Facilitated diffusion of glucose is the process by which D-glucose enters red blood cells; it is not impacted by insulin and is not ATP-dependent. The glycolytic route in RBCs uses 90% of the glucose, with the remaining 10% passing through the pentose shunt. Numerous changes in erythrocytes have been seen in diabetics, including lower membrane cholesterol and sialic acid levels, shortened life span, decreased deformability, and increased RBC aggregation (Habib and Othman 2012). All of these modifications may have a significant impact on the uptake of glucose by the erythrocytes, as demonstrated by the current data which showed that the percentage of erythrocyte glucose uptake in DM rats was 31.6% and in the healthy group was 37.61%. After addition of PHZ or its PHZ-SeNPs, these percentages rose to be 58.29% and 60.78%, respectively. Further, by activating 5’-adenosine monophosphate (AMP) kinase (AMPK), ADP has been shown to improve glucose uptake, insulin sensitivity and fatty acid oxidation (Kelly et al. 2006). According to our findings, diabetic-treated rats had higher serum ADP levels than diabetic untreated rats. This can lead one to suggest that the glucose uptake by RBCs in rats may follow the latter mechanism during lowering of their glucose levels. Furthermore, ADP adjusts fats metabolism and carbohydrates, increasing fatty acid oxidation and glycolysis while decreasing gluconeogenesis. For these reasons, the increase in ADP lowers blood insulin levels and fat storage. Furthermore, fatty acid oxidation rises and lipogenesis falls, helping to keep serum lipid levels from building up and thus reduce HOMA-IR and subsequent improvement in the quality of life in diabetic individuals.

PHZ is well known as a competitive inhibitor of sodium-glucose cotransporters (SGLT1 and SGLT2) in the intestine and kidney, red blood cells (RBCs) exclusively express GLUT1 as their glucose transporter and do not contain SGLT proteins. PHZ remains chemically stable and is not hydrolyzed to its aglycone metabolite phloretin, which is the known inhibitor of GLUT1 (Hytti et al. 2023). Therefore, the observed increase in RBC glucose uptake after PHZ treatment is unlikely to result from GLUT1 inhibition. Instead, PHZ may modulate membrane dynamics or transporter conformation, enhancing GLUT1-mediated facilitative diffusion of glucose across the RBC membrane (Ferté et al. 2021). Additionally, PHZ-SeNPs could further potentiate this effect by stabilizing PHZ, preventing its enzymatic degradation, and improving its interaction with cell membranes. Hence, PHZ and PHZ-SeNPs are proposed to indirectly enhance GLUT1-mediated glucose transport through altered membrane permeability and improved transporter efficiency, rather than acting as direct inhibitors.

STZ administration induces β-cell injury and functional impairment. In the early stage of experimental diabetes, this may trigger a compensatory increase in islet number and size. However, continued β-cell damage ultimately leads to islet degeneration and loss, depending on disease duration. The possible mechanism may involve production of reactive oxygen species and subsequent induction of inflammation (Koroglu et al. 2025). PHZ; an apple Polyphenolic, exert hypoglycemic effects and is a precursor of SGLT2 inhibitors. Its oral administration induces renal glycosuria by reducing the reabsorption of glucose from the glomerular filtrate. This cause elevation in urinary glucose excretion without hyperglycemia (Schulze et al. 2014). This mechanism is typically similar to that of the commercially used drug; dapagliflozin, does (Liu et al. 2020). These treatments cause improvement in the pathophysiological findings of the kidneys, liver and in pancreas, in the present study.

Because Se NPs are less toxic, need less dosage for improved reactivity, and have high absorption when compared to other Se oxidation states, they have several uses in medicinal processes (Wadhwani et al. 2016). In the present study, sodium selenite was combined with PHZ to form Se NPs-based delivery system for PHZ i.e. PHZ-SeNPs. The aim of this combination is to enhance the bioavailability of flavonoids. Selenium contributes to the regulation of glucose metabolism mainly through indirect mechanisms, such as enhancing antioxidant enzyme activity and modulating insulin signaling pathways (Casanova and Monleon 2023). It has been reported that Se can influence key enzymes in the insulin signaling cascade, such as hexokinase and p70-S6 kinase, thereby supporting glucose utilization and improving insulin sensitivity (Abdel Moneim et al. 2015). In the present study, Se NPs ameliorated hyperglycemia by improving HOMA-IR, HbA1c, and lipid profile levels, indicating enhanced insulin sensitivity rather than a direct insulin-mimetic effect.

Further, Se is a cofactor for some of the antioxidant enzymes that protect against inflammations, as the PHZ content of flavonoids do. This is not only confirming the anti-inflammatory effects of PHZ but also PHZ capping for the Se NPs. Such capping is also responsible for preventing liver lesions in rat’s models. Therefore, absence of these PHZ-SeNPs can not only damage the liver but also pancreas and kidneys (Ullah et al. 2021). The histopathological improvements in kidney, liver and pancreas after treatments necessitate the presence of antioxidant’s activities. In spite, PHZ-SeNPs induce more reduction in serum glucose levels and more enhancements in organs healing nearly to their normal physiology. Therefore, one can suggest that the flavonoids and phenolic contents of PHZ-SeNPs coat can add more to PHZ only in reducing blood glucose levels; possibly via the PHZ capping or their antioxidant properties. Confirming this, the treated diabetic groups; particularly those treated with PHZ-SeNPs, had higher mean blood levels of GSH, TAC, and catalase and SOD activity than the untreated group. Additionally, in diabetes-induced rats, the current study tried to clarify the hypoglycemic and anti-atherogenic effects of PHZ and its Se nanoparticles (PHZ-SeNPs).

Significant abnormalities of the blood biochemical markers associated with glucose metabolism were caused by diabetes in the current investigation. In comparison to diabetic rats that were not treated, the diabetic rats that received phlorizin or PHZ-SeNPs shown notable reductions in their mean fasting blood glucose level, insulin level, HbA1c level, and in the calculated HOMA-IR. Our results confirm those of Wang et al. (2019) who observed that diabetic rats had significantly increased fasting insulin, FBG levels and estimated HOMA-IR than normal rats (*p* < 0.01); a result which was expected and was the case in the present study.

The HOMA-IR reflects basal insulin resistance under fasting conditions. It provides insight into how the body’s tissues respond to circulating insulin and indicates chronic alterations in glucose-insulin homeostasis. It often refers to the lower sensitivity of human liver, muscle and adipose cells toward insulin, making it difficult for blood glucose to enter cells for metabolism and energy supply; thus, more insulin must be secreted by the pancreas for hyperinsulinemia to occur (Huang and Chen 2022). In another study involving a clinical trial, the serum insulin was respectively reduced after feeding diabetic rats with metformin and metformin–nano-selenium for 8 weeks, and the HOMA-IR dropped by 17.92 and 22.41%. It was postulated that the administration of selenium nanoparticles may induce a rise in selenium protein and result in the elevation of the sensitivity of the insulin signal transduction route. while for patients with type 2 diabetes mellitus, the serum insulin and HOMA-IR was lowered, following the intake of cinnamon for 3 months (Zare et al. 2019). The presence of phenolic compounds in cinnamon extract was found to increase phosphoinositide 3-kinase activity for the subsequent elevation of glucose transporter 4 expression and the attenuation of inhibiting glycogen synthase, leading to the promotion of glucose transportation within cells and increase in glycogen synthesis so that glucose utilization was raised (Imparl-Radosevich et al. 1998). Nevertheless, the increase in bioavailability through administration of nanoparticle may also play a vital role in enhancing the treatment efficiency of diabetes mellitus.

The oral glucose tolerance test (OGTT) is a procedure that determines whether a patient can use and store glucose normally. OGTT evaluates the dynamic glucose handling following a glucose load, reflecting both insulin secretion and peripheral glucose uptake in response to acute glycemic challenge. It captures the integrated response of the pancreas, liver, and peripheral tissues to a glucose load (Chuang et al. 2013). In clinical settings and animal trials, OGTT is frequently used as diagnostic tool for assaying the impairment in glucose tolerance (IGT). Likewise, the improvement of animals’ IGT is shown by the reduction of the elevated AUC. The OGTT is widely used in animal studies to evaluate the severity of diabetes and to examine the medications’ effects on the body’s ability to metabolize glucos.The geometric mean value used to measure blood glucose during an OGTT is called the AUC.According to a clinical trial, diabetic rats’ AUC was noticeably higher than that of normal glucose-tolerant individuals (Schulze et al. 2014). In our work, diabetic rats showed a considerable rise in AUC. Because of the reduction in its AUC, PHZ-SeNPs exhibit significant potency.

ADP is secreted by adipose tissue which has protective anti-inflammatory effect. Additionally, it improves insulin sensitivity in both people and obese animals (Khoramipour et al. 2021). Taking together, ADP plays a significant protective role against atherosclerosis as well as against diabetes (Achari and Jain 2017). Further, it aggressively controls blood sugar and regulates glucose tolerance (Takeda et al. 2012). Moreover, ADP promotes the oxidation of fatty acids, and subsequently minimizes TG accumulation (Hara et al. 2006). In our opinion, all of these participate in the modification of glucose homeostasis to protect against diabetic complications.

Rats treated with PHZ had non-significantly higher ADP mean levels and A/L ratios than rats in the diabetic group. When the treatment was compared with the healthy rats, the difference was incredibly significant. Additionally, when compared with diabetes-induces rats, the mean ADP level in the serum of PHZ-SeNPs diabetic treated rats was considerably elevated (*p* < 0.001). Similarly, sodium-glucose cotransporter 2 inhibitors (SGLT2i) are a new family of oral hypoglycemic medications which was used in conjunction with lifestyles modification to treat human metabolic syndrome (HMS), according to Menzies-Gow and Knowles (2025).

Under particular metabolic circumstances, leptin modifies the consumption of fuels by acting either autocrinely or paracrinely. Leptin release is further enhanced by long-term insulin exposure. It was discovered that the concentration of leptin in adipose tissue may be greater than that in the blood. Furthermore, leptin reflects adiposity, increases lipolysis and promotes insulin resistance while lowering fat synthesis in experimental animals’ adipose tissue metabolism (Caron et al. 2018). When compared to the healthy rats in the current investigation, the mean leptin level in the sera of the diabetic rats was higher. Following PHZ treatment, the mean level was considerably decline compared with the control level (*P* < 0.001).

Also, the disturbed lipid profile of the diabetic rats was restored after treatment with PHZ-SeNPs. This is because leptin is the key player in the regulation of lipids mobilization, their oxidation and their synthesis (Koltes et al. 2017). According to Zeng et al. (2015), this control of lipid metabolism in adipose tissue occurs through receptor interaction and sympathetic innervations, which include catecholamine release and β-adrenergic receptor (βAR) activation. Unfortunately, catecholamine’s were not evaluated in this study. Therefore, to understand the pathogenesis of T2D, leptin measurement must be selected (Picó et al. 2022), as demonstrated in the current investigation, where sera cholesterol, T.G. and LDL levels were decreased after treatment with PHZ-SeNPs compared to those of the non-treated diabetic rats. Conversely, HDL levels were increased to help in the miscibility of LDL-cholesterol, thus retard atherosclerosis.

Our results agree with that of Zhang et al. (2025) who observed reduction in fasting insulin levels, and modulation of ADP levels (reduced leptin and increased in ADP) in high-fat diet-induced mice treated by berberine and evodiamine. The leptin/adiponectin (LAR) and adiponectin/leptin (A/L) ratios serve as more sensitive indicators of metabolic dysfunction than either adipokine alone. A higher LAR or lower A/L ratio indicates a shift toward insulin resistance, impaired glucose metabolism, and systemic inflammation (Inoue et al. 2006). Also, Inoue et al. (2006) suggest that A/L ratio might be more useful than HOMA-IR to accurately assess insulin resistance in subjects without hyperglycemia. In our opinion, LAR is more correlated with the diabetic marker namely; HbA1c, HOMA-IR, FBG and insulin. Thus, it can be used as a diabetic marker rather than A/L ratio.

Concerning lipids metabolism, the mean level of T.G., cholesterol, LDL cholesterol, VLDL and total lipid in diabetic rats-treated with PHZ or PHZ-SeNPs were significantly lower (*p* < 0.001). Although, the mean HDL-cholesterol level was significant increase if compared with diabetes-induced rats (*p* < 0.01). This increase facilitates lipolysis. The latter prevent atherosclerosis and prevent induction of fatty liver which was also the case in our investigation according to the reduction of liver bilirubin and liver enzymes. According to Londzin et al. (2018) showed that administering PHZ (20 mg/kg) didn’t favorably affect while administering (50 mg/kg) enhanced the parameters of lipid metabolism.

These results agree with Zhang et al. (2025) who observed lowered serum TC, LDL-C, and TG levels, while simultaneously increase in the concentrations of HDL-C in high-fat diet-induced mice treated by berberine as well as evodiamine. Also, with Abdel Moneim et al. (2015) and Najafian et al. (2012). In their results the animals which were either treated with Se NPs alone or with insulin showed significantly decrease in FBG levels after 28 days of the start of treatment when measured day after day. These results suggested that Se NPs can alleviate hyperglycemia and hyperlipidemia in diabetes- induced rats, possibly via eliciting insulin-mimicking activity.

As was expected, uncontrolled diabetes is an inflammatory disease which can disrupt many of the organ’s functions including liver and kidney. With respect to the liver functions transaminases activities were elevated after diabetogenesis. After treatment of such disease with insulin, PHZ and PHZ-SeNPs were highly significant decrease but the mean levels of albumin and total proteins extremely significantly increased when compared to that of the diabetic groups. These outcomes concurred with the findings of Abdel Moneim et al. (2015). To confirm, Dkhil et al. (2014) showed that ALT and AST are very reliable biomarkers and have been strongly associated with liver damage and toxicity. According to Ghosh et al. (2013), the elevate in transaminase activities in sera of diabetes rats may be primarily caused by the enzymes leaking into the bloodstream from the liver cytosol as a result of hepatic injury linked to STZ inducer, oxidative stress, and lipid accumulation that accompany the induction of diabetes which is the situation in this investigation, where the metabolism of lipids was changed both before and after therapy.

A large portion of research on the common consequences of diabetes has focused on the kidney. Since nephropathy, micro-vascular disease, and retinopathy are markers of diabetic complications (Abdel Moneim et al. 2015). Creatinine, urea and uric acid were estimated for the animals in this study. In this study derangements of the latter markers in diabetic rats were observed. After treatment, these markers were modified indicating enhancement of the kidney tissues as was the histopathological findings of the kidney tell us. These results were in agreement with those obtained by Abdel Moneim et al. (2015).

Currently, diabetes-induced rats which were treated with insulin, PHZ, or PHZ-SeNPs showed highly substantial decreases in mean MDA and NO levels. Conversely, when compared to the diabetic groups, the mean levels of GSH, CAT, TAC, and SOD were significantly higher. These results match those of Saad et al. (2017), who demonstrated that diabetic rats had much higher MDA levels. These confirmed that both type 1 and type 2 diabetic rats had significantly higher lipid peroxidation levels than the healthy controls. Therefore, the concurrent deterioration of organs tissues may follow oxidative stress mediated mechanisms. Antioxidant defenses may also be compromised by drop in GSH levels. This is because oxidative damage is a characteristic of the early and late phases of diabetes. Our results confirm those of Azeem et al. (2023) who confirmed that the treatments with Se NPs-mediated Citrus paradisi peel extract prevented bisphenol-induced liver in male rats possibly by increasing CAT and SOD activities as well as the mean GSH level.

In the current work, the rise in the level of liver glycogen, main storage form of glucose, in rats treated with PHZ-SeNPs may be due to stimulation of glycogen synthase activity. The latter, in turn increases liver glycogenesis. Under typical physiological circumstances, PHZ-SeNPs stimulate insulin secretion and thus promotes glycogen deposition by further activation of glycogen synthase and inhibiting glycogen phosphorylase (Abdel Moneim et al. 2015). Such deposition is mediated also by Se which is responsible for preventing liver lesions in rat’s models and for the antioxidant effect of PHZ-SeNPs. Therefore, Se deficiency can not only damage the liver but also pancreas and kidneys (Ullah et al. 2021). Also, providing Se as a cofactor for some antioxidant enzyme, one can confirm the protective effect of Se against liver, pancreas and kidneys damages (Drake 2006).

The observed increase in β-cell islet number and size in the diabetic group likely reflects an early compensatory response to insulin resistance, consistent with previous reports describing β-cell hyperplasia during the initial stages of type II diabetes mellitus (Alejandro et al. 2015). Such adaptive changes precede β-cell exhaustion, atrophy, and amyloid deposition, which typically require prolonged disease duration. Therefore, the absence of advanced degenerative pancreatic changes in the present study is consistent with the experimental timeframe and the metabolic nature of type II diabetes. The reduction in islet number and size in treated groups likely reflects attenuation of compensatory β-cell hyperplasia following metabolic improvement rather than β-cell loss. Future studies incorporating immunohistochemical markers, including chromogranin A and synaptophysin, may provide additional histological insights and further strengthen the assessment of pancreatic endocrine alterations.

In T2D, FBG, HbA1c, HOMA-IR, insulin, Leptin, LAR and lipids profile except that of HDL and cholesterol were negatively correlated with serum ADP levels. ADP may influence insulin sensitivity by reducing adipose tissue T.G. and controlling the insulin signaling pathway, which increases the oxidation of fatty acids and, consequently, energy expenditure. As a result, ADP was thought to be among the most reliable indicators of T2D. ADP impact on insulin sensitivity was initially documented in mice (Gupta et al. 2018). These outcomes match with those of Lee et al. (2009), who demonstrated that, individuals with T2D and metabolic syndrome had higher leptin values and A/L ratio but lower levels of total ADP in their blood. These results support the negative correlation between the latter and HOMA-IR. Conversely, there was a positive correlation between HOMA-IR and leptin. ADP and HDL levels, on the other hand, exhibited a positive and substantial connection with the A/L ratio. In non-diabetic white people, Finucane et al. (2009) found a robust and statistically significant positive connection between fasting insulin and the LAR. This is due to the fact that hypertrophic adipocytes release less adiponectin and more leptin. Therefore, the LAR has been suggested as a potentially helpful indicator of vascular risk and insulin resistance. These findings shed additional light on the pathophysiology of insulin resistance and the crucial role that adipocytes malfunction plays.

In our study, a significant relationship was observed between insulin resistance (HOMA-IR) and adipokine ratios, supporting the interconnection between adipose tissue dysfunction and impaired glucose homeostasis. HOMA-IR showed a strong positive correlation with the LAR (*r* = 0.68, *p* < 0.001) and a significant negative correlation with the A/L ratio (*r* = − 0.45, *p* < 0.01). These findings indicate that higher leptin levels relative to adiponectin are associated with increased insulin resistance, whereas elevated adiponectin relative to leptin reflects improved insulin sensitivity. Treatment with PHZ and PHZ-SeNPs reduced HOMA-IR and normalized these adipokine ratios, suggesting that their antidiabetic effect may involve modulation of adipose-derived hormones in addition to improved glucose metabolism and oxidative balance. Thus, it can be concluded that HOMA-IR still a more reliable indicator of insulin resistance than LAR. These findings were similar to the study of Jung et al. (2010) who showed that HOMA-IR had a significant negative correlation with A/L ratio in subjects with T2D. Also, in multiple regression analysis, HOMA-IR and A/L ratio affected each other and were significant predictors for each other, suggesting that A/L ratio might be used as an insulin resistance marker in healthy males (Jung et al. 2010).

## Conclusion

In this study, PHZ-SeNPs were successfully green-synthesized using PHZ and sodium selenite and characterized spectroscopically by UV-Vis, FT-IR, TEM, and zeta potential analyses, confirming the formation of selenium nanoparticles capped with PHZ. The capping by PHZ played a crucial role in preventing nanoparticle aggregation and enhancing their stability and sustained release potential.

Experimentally, the administration of PHZ-SeNPs to diabetic rats markedly improved glycemic control and lipid metabolism, as indicated by reduced fasting blood glucose, HbA1c, and HOMA-IR values, along with increased insulin levels. These effects were accompanied by restoration of antioxidant balance and protection of hepatic, pancreatic, and renal tissues. The regulatory roles of leptin and adiponectin further suggest improved lipid handling and insulin sensitivity.

The beneficial metabolic outcomes observed may be attributed to the combined antioxidant activity of PHZ and Se, together with enhanced insulin signaling rather than a direct insulin-mimetic effect. Collectively, these findings propose PHZ-SeNPs as a promising adjuvant therapeutic candidate for the management of diabetes and its related hepatic and renal complications.

## Supplementary Information

Below is the link to the electronic supplementary material.

Supplementary Material 1

Supplementary Material 2

## Data Availability

All data generated or analyzed during this study are included in this manuscript.

## Abbreviations

STZ: Streptozotocin
PHZ-SeNPs: Phlorizin-selenium nanoparticles
TEM: Transmission electron microscopy
FT-IR: Fourier transform infrared spectroscopy
DPPH: 2, 2- diphenyl-1-picryl hydrazyl-hydrate free radicals
i.p.: Intraperitoneal
FBG: Fasting blood glucose
HbA1c: Glycosylated hemoglobin
HOMA-IR: Homeostatic model assessment of insulin resistance
OGTT: Oral glucose tolerance test
ADP: Adiponectin
LAR: Leptin/Adiponectin ratio
HDL: High density lipoprotein
