# Supplementary material for: In vivo and in vitro antidiabetic effects of phlorizin and its green-synthesized phlorizin-selenium nanoparticles in male rats: mechanistic involvements and nanoparticles characterization
Source: Bioresour Bioprocess. 2026 Apr 27;13(1):58. doi: 10.1186/s40643-026-01034-3 (PMC13121686; doi:10.1186/s40643-026-01034-3)
Supplement: Supplementary file 1 — Supplementary Material 1 [file 40643_2026_1034_MOESM1_ESM.pdf]

Table S1 . Semi-quantitative histopathological assessment of pancreatic tissue

Group	Presence of Pancreatic Tissue	Presence of B-Islets	Scoring (No. of islets / 10 HPFs; No. of cells per islet)
**Control**	Present	Present	4 foci (5–10 cells per islet)
**DM**	Present	Present	5 foci (50–100 cells per islet)
**Insulin**	Present	present	1 focus (6 cells per islet)
**PH -treated**	Present	Present	1 focus (7 cells per islet)
**Nano -Selenium treated**	Present	Present	1 focus (10 cells per islet)

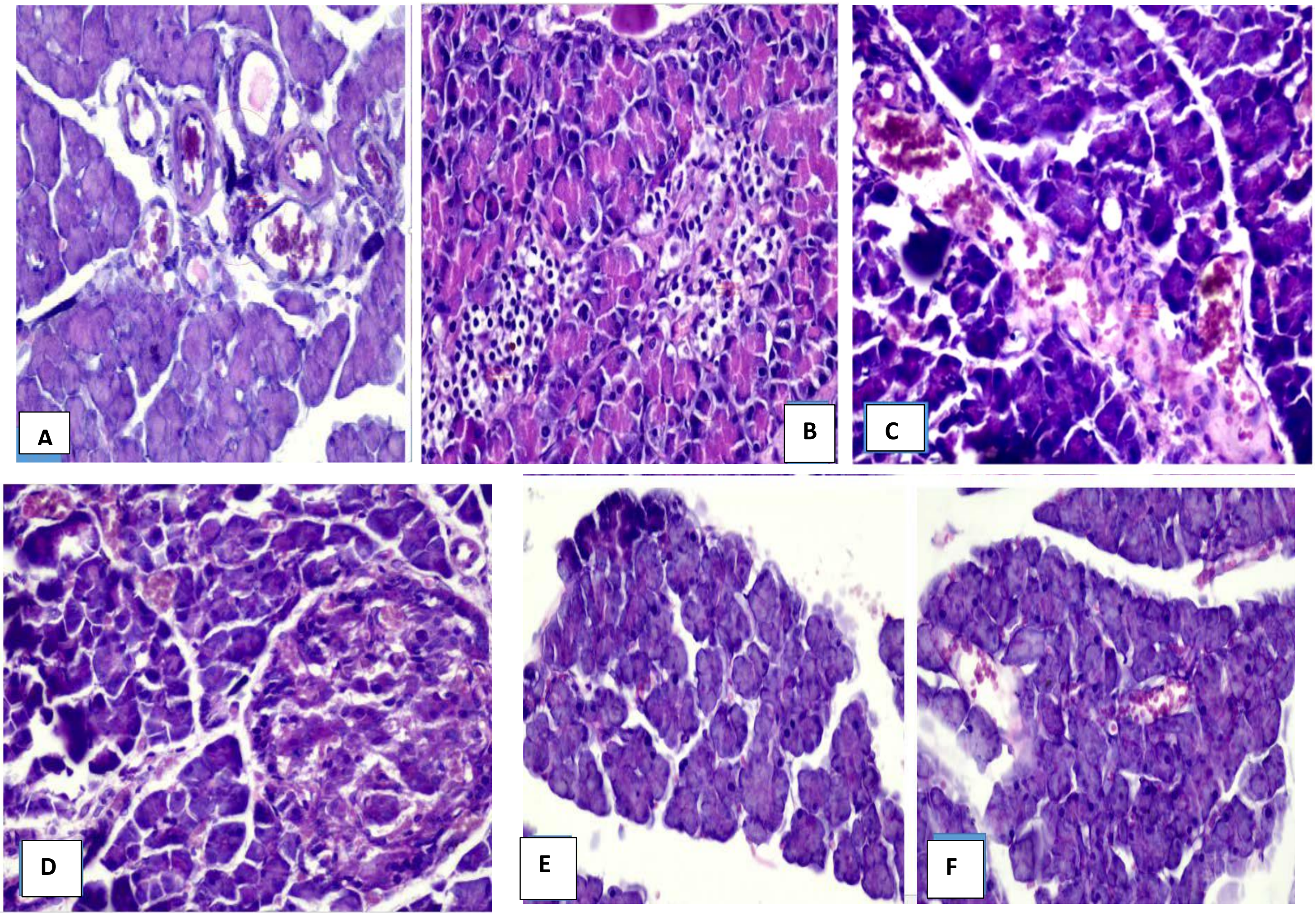

Figure S1: Representative photomicrographs of pancreatic tissue stained with H&E. **A.** Representative photomicrographs of pancreatic tissue from the Control group showing few B-cell islets arranged in a perisinusoidal pattern, with four foci per 10 HPFs and 5–10 cells per islet. **B.** Representative photomicrographs of pancreatic tissue from the DM group demonstrating numerous B-cell islets arranged in a perisinusoidal pattern, with five foci per 10 HPFs and 50–100 cells per islet. **C.** Representative photomicrographs of pancreatic tissue from the insulin group showing the presence of B-cell islets, with one focus per 10 HPFs and approximately 6 cells per islet. **D.** Representative photomicrographs of pancreatic tissue from the Nano group showing few B-cell islets arranged in a perisinusoidal pattern with one focus per 10 HPFs and approximately 10 cells per islet.**E &F .** Representative photomicrographs of pancreatic tissue from the PH group showing few B-cell islets arranged in a perisinusoidal pattern with one focus per 10 HPFs and approximately 7 cells per islet (×400 magnification).
