# Supplementary figures and images for: In vivo and in vitro antidiabetic effects of phlorizin and its green-synthesized phlorizin-selenium nanoparticles in male rats: mechanistic involvements and nanoparticles characterization
Source: Bioresour Bioprocess. 2026 Apr 27;13(1):58. doi: 10.1186/s40643-026-01034-3 (PMC13121686; doi:10.1186/s40643-026-01034-3)

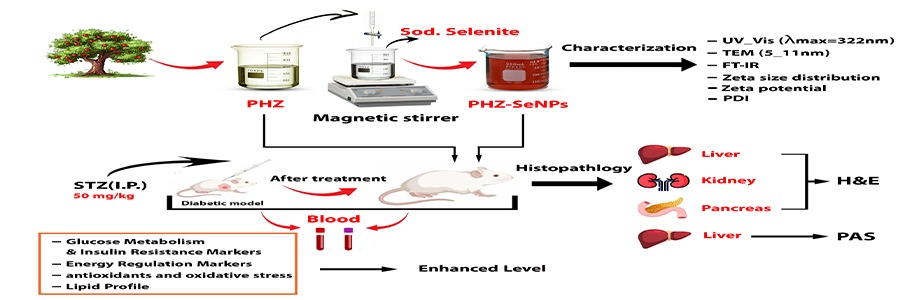

Supplement: Supplementary file 2 — Supplementary Material 2 [file 40643_2026_1034_MOESM2_ESM.jpeg]
